# Data reduction for SVM training using density-based border identification

**DOI:** 10.1371/journal.pone.0300641

**Published:** 2024-04-03

**Authors:** Mohammed Shalaby, Mohamed Farouk, Hatem A. Khater

**Affiliations:** 1 Department of Computer Science, College of Computing and Information Technology, Arab Academy of Science, Technology & Maritime Transport, Alexandria, Egypt; 2 Mechatronics Engineering Department, Faculty of Engineering, Horus University Egypt, New Damietta, Egypt; Chunghwa Telecom Co. Ltd., TAIWAN

## Abstract

Numerous classification and regression problems have extensively used Support Vector Machines (SVMs). However, the SVM approach is less practical for large datasets because of its processing cost. This is primarily due to the requirement of optimizing a quadratic programming problem to determine the decision boundary during training. As a result, methods for selecting data instances that have a better likelihood of being chosen as support vectors by the SVM algorithm have been developed to help minimize the bulk of training data. This paper presents a density-based method, called Density-based Border Identification (DBI), in addition to four different variations of the method, for the lessening of the SVM training data through the extraction of a layer of border instances. For higher-dimensional datasets, the extraction is performed on lower-dimensional embeddings obtained by Uniform Manifold Approximation and Projection (UMAP), and the resulting subset can be repetitively used for SVM training in higher dimensions. Experimental findings on different datasets, such as Banana, USPS, and Adult9a, have shown that the best-performing variations of the proposed method effectively reduced the size of the training data and achieved acceptable training and prediction speedups while maintaining an adequate classification accuracy compared to training on the original dataset. These results, as well as comparisons to a selection of related state-of-the-art methods from the literature, such as Border Point extraction based on Locality-Sensitive Hashing (BPLSH), Clustering-Based Convex Hull (CBCH), and Shell Extraction (SE), suggest that our proposed methods are effective and potentially useful.

## 1 Introduction

In recent years, large datasets have become increasingly prevalent in various domains, posing challenges for traditional machine learning algorithms due to their computational complexity and memory requirements. These challenges motivated the creation of various methods and algorithms that aim at minimizing the bulk of the training data to speed up training the models without compromising their accuracy. Data reduction techniques have been extensively researched and implemented to address this issue. These techniques can be broadly categorized into dimensionality reduction, cardinality reduction, and numerosity reduction techniques [1]. Dimensionality reduction, including feature selection and extraction techniques, focuses on identifying and selecting the most informative attributes from a dataset [2]. Cardinality reduction techniques, such as binning and discretization, transform the original data into a reduced representation [1, 3]. Numerosity reduction, including instance selection or sampling techniques alternatively, aims at choosing a representative subcategory of a dataset by removing redundant and noisy data points [4].

Support Vector Machines (SVMs) are powerful and effective machine learning algorithms that have shown excellent performance in various tasks. The algorithm tries to locate a hyperplane that divides data points of various classes with the greatest possible margin by optimizing a quadratic programming problem [5]. This problem is computationally expensive and can be prohibitive for applying the algorithm to large datasets. However, it was found that only a subset of the training data points, called *support vectors* (SVs), contribute to the final decision boundary, while the remaining data points can be considered redundant and thus potentially removed [6]. As a result, instance selection techniques have been developed to identify data points that the SVM algorithm is more likely to choose as support vectors. These techniques frequently rely on the assumption that these support vector candidates are located near the decision boundary [7]. Many research efforts have been directed towards developing instance selection algorithms for SVM training data reduction. In this part, a few of the most important techniques are briefly discussed. Here is a brief summary of the most pertinent connected work.

Barros de Almeida et al. [8] used k-means to divide the dataset into small clusters. Clusters consisting exclusively of data points with identical class labels were discarded and substituted with their centroid. Clusters that contained data points from various categories were retained. This method is affected by the distribution of the dataset and the presence of noisy data points.

Koggalage and Halgamuge [9] used a variable radius around cluster centers to recognize crisp regions that comprise data points of the same category. To avoid removing potential support vectors in crisp clusters near the decision boundary, a variable safety margin is applied, and only data points that lie between cluster centers and the safety margin are removed.

Wang et al. [10] introduced the K-means SVM (KM-SVM) algorithm, which aimed at reducing the inference time of the SVM classifier by minimizing the number of identified SVs. They used k-means to condense the data into cluster centers, which were then used for searching the input parameter space for a good combination of the regularization parameter (C), gamma, and compression rate parameters that yielded fewer support vectors. Their algorithm showed promising results; however, it did not consider the respective contributions of distinct cluster centers in determining the decision boundary.

In their study, Bang and Jhun [11] proposed enhancements to the KM-SVM approach to account for the significance of different cluster centers by introducing a weight depending on the number of data points within each cluster during the optimization process. Additionally, the authors suggested a weighting strategy for imbalanced datasets. They also proposed an additional process for recovering data points that are in proximity to the SVs. These salvaged data points are then used to reconstruct the SVM classifier. This improves the precision of the selected model, but, as a consequence of the additional processes and the increase in the number of SVs, training and inference times are relatively prolonged.

Demir and Erturk [12] applied k-means clustering separately to each class to obtain cluster centroids. The number of clusters used for k-means is determined based on the number of data points in the class. An SVM classifier is then learned on the set of centroids. Data points of clusters whose centroid is identified to be a support vector, in addition to the centroids themselves, comprise the final reduced training set.

Shen et al. [13] used a two-stage approach; first, the dataset is partitioned using k-means. The resulting clusters are further divided into sub-clusters based on class labels. The centroids of the clusters are employed to train an SVM to obtain an approximate hyperplane, which is then used to discard clusters that are far away from it. For the second stage, the authors proposed the Fast Iteration of FDR (FIFDR) algorithm, in which the Fisher Discriminant Ratio (FDR) is applied using distance densities of points to the centroids to obtain a boundary between the dense area of data points near the centroids and the outer sparse area of data points. Points within the boundary are removed.

Lui et al. [14] proposed the Shell Extraction (SE) algorithm, in which, first, the geometric centroid of each class is calculated. Then, a certain radius around each centroid defines a reduction sphere, and data points within the sphere are removed. The radius is calculated based on the average distance between the centroid and the data points of the class and a user-defined parameter. This process is iteratively applied to the remaining data points. For each iteration, the new centroids are calculated and the reduction spheres are expanded. The algorithm terminates when the number of retained data points is less than a user-defined threshold. The algorithm assumes that the classes are spherical. Furthermore, its output is dependent on parameters controlling the radii of the reduction spheres and the rate of their expansion.

In the clustering-based convex hull (CBCH) algorithm introduced by Biranzhandi and Youn [15], the dataset is partitioned by k-means into clusters. All the data points of clusters containing different class labels are preserved. For clusters containing the same class label, the convex hulls are calculated for each cluster, and only the data points at their vertices are retained while the rest of the data points are removed. The procedure’s efficiency is dependent on the number of clusters and their preliminary centroids.

Aslani and Seipel [16] introduced the border point extraction based on locality-sensitive hashing (BPLSH) algorithm, in which data points are allocated to buckets using a collection of hash function families based on locality-sensitive hashing (LSH). A similarity index, expressed as the count of common buckets for a pair of points across all families of hashing functions, is used to quantify the closeness between points. Samples with quite close neighbors of an opposite class are considered border data points and are retained. Only one representative instance is preserved in a homogeneous region. Similar to other methods using partitioning of the dataset, performance is affected by the granularity of partitions.

In his approach, Gaffari [17] proposed a method aiming to reduce both the data set size and boundary complexity. Based on the neighborhood information of each data point, harmful data points are removed. A harmful point has a class label that is different from the dominant class label of its neighbors. Outlier points, which are not in the neighborhood of other points, are also removed. Boundary points are then determined based on being one of the mutually closest pairs of points of different classes. Then, from the remaining points, those whose closest point of a different class is still not selected are also added to the boundary points. The remaining non-boundary points are condensed into cluster centers using hierarchical clustering. Those representatives, combined with the boundary points, form the reduced dataset.

The previously mentioned related work has shown that instance selection can be employed to reduce the bulk of the training set without significantly affecting the accuracy of the SVM classifier. However, most of these methods are based on clustering the whole dataset into partitions and then detecting samples in proximity to the decision boundary by checking for the occurrence of multi-class samples in the clusters. This method is affected by the distribution of the dataset as well as the occurrence of anomalies in the dataset. Moreover, it is also sensitive to the granularity of the clusters and the initialization of the centroids. For instance, as the granularity of clusters increases, the majority of clusters will likely contain only a single class label. In contrast, with lower granularity, it will be more likely that more than one class label will be included in most of the clusters, thereby decreasing the accuracy of their selection. Furthermore, most of the techniques mentioned do not have control over the size of the reduced dataset.

The main aim of this research is to identify a layer of data points at the borders of different classes, as they most likely include data points to be selected as support vectors by the SVM algorithm. Retaining such a layer of data points and using it for training can enhance the training efficiency of the SVM algorithm while not significantly compromising classification accuracy. This is especially important when applying SVM to large-scale datasets.

The key contributions of this study are as follows:

- A novel density-based method for SVM training data reduction, called Density-based Border Identification (DBI), is proposed, which utilizes density-based techniques to identify a layer of border data points that are likely to include support vectors identified by the SVM algorithm.
- Four variations of the DBI method are also proposed. These variations either extend the DBI techniques or replace them with different strategies for the selection of data points to achieve improved performance.
- An approach for speeding up the training of the SVM classifier in higher dimensions is introduced. This approach is based on applying instance selection in lower-dimensional embeddings before mapping the reduced dataset back to the original higher dimensionality for training, which allows the use of the same reduced dataset for multiple training sessions.
- A modified implementation of k-fold cross-validation is proposed, which is tailored for training the SVM on a subset of the dataset with a different distribution.
- A balanced evaluation of the proposed methods is presented by simultaneously considering multiple objectives, namely accuracy, training speedup, and testing speedup.

The rest of the paper is organized as follows: In Section 2, a brief background is presented for the SVM and density-based techniques. We describe the suggested algorithm, the SVM training approach, and the four variations of the algorithm in Section 3. Section 4 comprises the experimental setup, evaluation methodology, and experimental results. A general discussion of the main findings is presented in Section 5. Finally, Section 6 concludes the research.

For reference, a list of the acronyms used is available in S2 File.

## 2 Background

This section offers a concise background on the SVM algorithm and the DBSCAN (Density-Based Spatial Clustering of Applications with Noise) clustering algorithm.

### 2.1 Support vector machines

**Fig 1:**
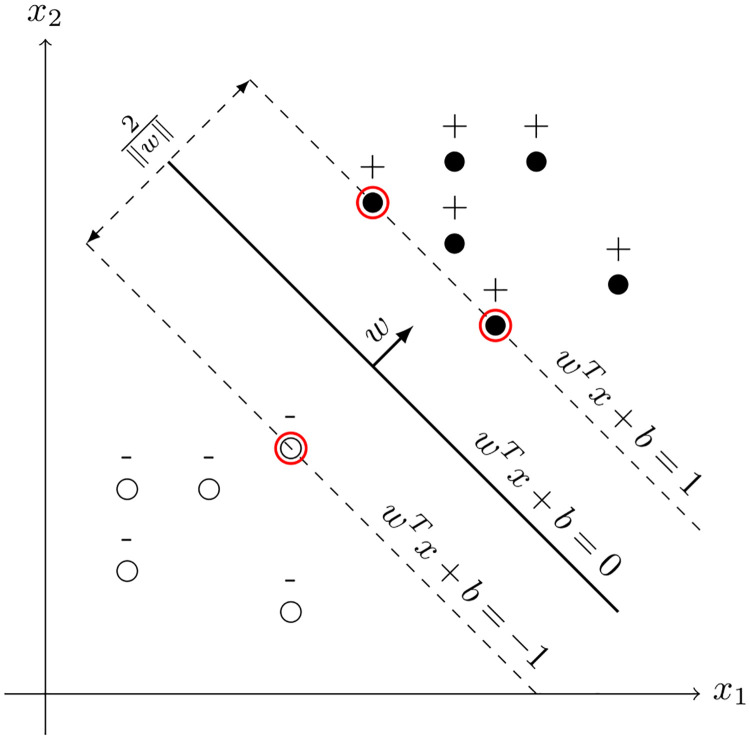
Separating hyperplanes in the linearly separable case. The hyperplane is shown as a solid line, the margins as dashed lines, and the support vectors are enclosed in red circles.

The SVM algorithm [18] is a supervised learning algorithm that can be utilized to solve classification and regression problems. It is based on the idea of locating the optimal hyperplane that divides the data points into distinct classes. As depicted in Fig 1, the hyperplane is defined by the equation:

$$\begin{array}{c} w^{T}x+b=0 \end{array}$$ (1)

where *w* is the normal vector to the hyperplane and *b* is the bias term. The distance between a data point *x* and the hyperplane is given by:

$$\begin{array}{c} d=\frac{|w^{T}x+b|}{\left||w|\right|} \end{array}$$ (2)

The aim of the SVM algorithm is to maximize the margin between the hyperplane and the nearest data points, thus enhancing its generalization ability. These points are denoted as support vectors. The SVM algorithm can be expressed as the following quadratic programming problem:

$$\begin{array}{c} \begin{array}{cccc} & \underset{w,b,\xi }{\text{minimize}} & & \frac{1}{2}{\left||w|\right|}^{2}+C\sum_{i=1}^{n}\xi_{i}\\ & \text{subject}\;\text{to} & & y_{i}\left(w^{T}x_{i}+b\right)\geq 1-\xi_{i},\forall i=1,\ldots ,n\\ & & & \xi_{i}\geq 0,\forall i=1,\ldots ,n \end{array} \end{array}$$ (3)

where *C* is a regularization parameter and *ξ*_*i*_ is a slack variable that allows some data points to be misclassified.

The previous formulation of the optimization problem has a quadratic objective function and linear constraints. Consequently, it is a convex optimization problem that can be efficiently solved using the Lagrangian dual formulation. Since this formulation of the problem is convex, meaning that its objective function is quadratic and its constraints are linear, it is illegible for the Lagrangian dual formulation.

The Lagrangian dual formulation of the SVM optimization problem is given by:

$$\begin{array}{c} \begin{array}{cccc} & \underset{\alpha }{\text{maximize}} & & \sum_{i=1}^{n}\alpha_{i}-\frac{1}{2}\sum_{i=1}^{n}\sum_{j=1}^{n}\alpha_{i}\alpha_{j}y_{i}y_{j}x_{i}^{T}x_{j}\\ & \text{subject}\;\text{to} & & \sum_{i=1}^{n}\alpha_{i}y_{i}=0\\ & & & 0\leq \alpha_{i}\leq C,\forall i=1,\ldots ,n \end{array} \end{array}$$ (4)

where *α* is a vector of Lagrange multipliers, with each multiplier corresponding to a data point. The solution to the dual optimization problem determines the optimal values of the Lagrange multipliers *α*. In turn, the optimal values of *w* and *b* can be calculated as follows:

$$\begin{array}{c} \begin{array}{cc} w & =\sum_{i=1}^{n}\alpha_{i}y_{i}x_{i}\\ b & =y_{i}-\sum_{i=1}^{n}\alpha_{i}y_{i}x_{i}^{T}x_{j} \end{array} \end{array}$$ (5)

Eq 5 demonstrates that the optimal values of *w* and *b* depend only on points with nonzero Lagrange multipliers *α*_*i*_. These are known as support vectors. The following decision function can then be used to classify unseen data points:

$$\begin{array}{c} f\left(x\right)=sign\left(\sum_{i=1}^{n}\alpha_{i}y_{i}x_{i}^{T}x_{j}+b\right) \end{array}$$ (6)

Using the kernel trick, the SVM algorithm can be extended to non-linearly separable data. The kernel trick is based on mapping the data points to a higher-dimensional space in which they can be linearly separated. Without explicitly calculating the mapping function *φ*, the kernel function *K*(*x*_*i*_, *x*_*j*_) = *φ*(*x*_*i*_)^*T*^*φ*(*x*_*j*_) is used to compute the dot product of the mapped data points. The linear kernel, the polynomial kernel, and the radial basis function (RBF) kernel are the most frequently employed kernel functions. The equation $K\left(x_{i},x_{j}\right)=x_{i}^{T}x_{j}$ denotes the linear kernel. $K\left(x_{i},x_{j}\right)={\left(x_{i}^{T}x_{j}+c\right)}^{d}$ is the definition of the polynomial kernel, where *c* is a constant and *d* is the degree of the polynomial. The RBF kernel is introduced as *K*(*x*_*i*_, *x*_*j*_) = exp(−*γ*||*x*_*i*_ − *x*_*j*_||^2^) where *γ* is a constant [19].

The SVM algorithm is designed to solve binary classification problems, but it can be adapted to handle multi-class classification problems using the *one-versus-all* strategy. In this method, a separate SVM classifier is trained for each class, and the data point is allocated to the class with the maximum score. A data point’s score is quantified by the separation between the data point and the SVM classifier’s hyperplane [20]. *One-versus-one* is another approach, according to which a distinct SVM classifier is trained for every pair of classes. The data point is allocated to the class with the maximum number of votes. Since the number of SVM classifiers is quadratic in the number of classes, the one-versus-one technique is computationally more costly than the one-versus-all method. Nonetheless, it is more accurate than the one-versus-all approach because, unlike the one-versus-all approach, it does not introduce the class imbalance problem [21]. In this study, the one-versus-one approach is used.

### 2.2 Density-based clustering and DBSCAN

Our density-based approach is inspired by the concepts of the density-based clustering algorithm DBSCAN [22]. Three types of points are distinguished by the DBSCAN algorithm: core, border, and outlier. *Core points* are those with a minimum number of neighboring points (*minPts*) within a specified radius (*ε*). *Border points* lie within the radius of a core point but lack sufficient neighbors to be considered core points. *Outlier points* are not reachable from any of the core points within the specified radius.

Let *x* and *y* be two arbitrary data points. We say that *x* is *directly density-reachable* from *y* if *x* is within the *ε*-neighborhood of *y* and *y* is a core point, which means it has at least *minPts* neighbors within a distance of *ε*. *x* is said to be *density-reachable* from *y* if there exists a sequence of data points *z*_1_, …, *z*_*n*_ such that *z*_1_ = *y*, *z*_*n*_ = *x* and *z*_*i*+1_ is directly density-reachable from *z*_*i*_ for *i* = 1, …, *n* − 1. *x* is *density-connected* to *y* if there exists a data point *z* from which both *x* and *y* are density-reachable.

The procedure begins by choosing an arbitrary point *p* and determining all its *ε*-neighbors. If *p* has at least *minPts* neighbors, it is considered a core point; otherwise, it is an outlier. If *p* is a core point, a new cluster is formed with *p* and all its neighbors added. Then, the cluster expands by including the neighbors of the core points that were added to the cluster. This procedure repeats until all density-connected points are added. The algorithm then chooses another unvisited point and repeats the process. The algorithm terminates when all points have been visited [23].

## 3 Proposed method

**Fig 2:**
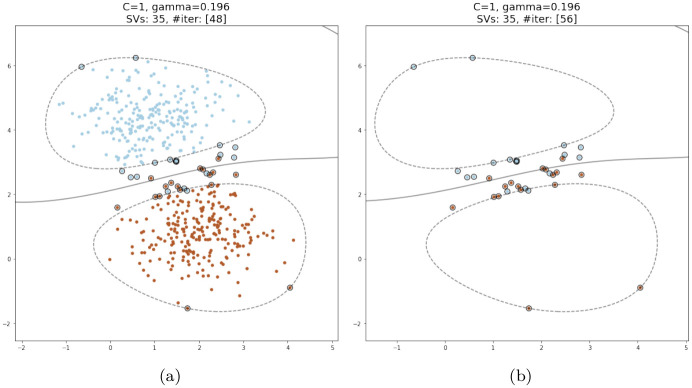
Training an SVM classifier only on support vectors compared to that trained on the whole dataset. The decision boundaries are shown as solid lines, the margins as dotted lines, and the support vectors are enclosed in circles. (a) A classifier trained on the entire dataset. (b) A classifier trained only on support vectors identified earlier by an SVM classifier trained on the whole dataset.

Most instance selection algorithms for reducing training data in SVM have the target of identifying a subset of the dataset that is most likely to contain the support vectors. This is justified by considering that the SVM classifier calculates the decision function using only the support vectors identified through the learning process. The rest of the training data is discarded at the end of the training phase. This observation suggests that other subsets of the training dataset exist with specific distributions and sufficient data points to guide the SVM algorithm in identifying support vectors that would produce a decision function for the SVM classifier that is comparable to what would be obtained using the original full dataset. The set of SVs extracted by the classifier trained on the entire dataset can be regarded as one such subset. Knowing the support vectors in advance is practically impossible without training the SVM classifier, which makes this only a hypothetical case. However, it was observed that training the SVM solely on these data points would result in the identification of nearly all of them as support vectors. Due to the elimination of all data points other than support vectors, the distributions of the reduced subset and the original dataset would differ significantly. Interestingly, experimentation has shown that the resulting decision boundaries are almost identical to those constructed by the SVM trained on the whole dataset, given that the same kernel function and hyperparameters are used, as illustrated in Fig 2.

In this work, we offer a density-based approach for instance selection, called Density-based Border Identification (DBI), in which density-based techniques are applied in a supervised manner. The neighborhoods of the data points of each class are analyzed separately, and a score is assigned to each data point according to the number of high-density neighbors (i.e., core points). These scores are then used to guide the splitting of the data points within each class into core, border, and outlier points. Outliers and core points are discarded, while border points are retained. The collective union of the border points of all classes comprises the final reduced training set. A user-defined *ratio* parameter controls the number of retained data points. DBI uses a similar method to DBSCAN to identify a layer of border points for each class. The main differences are that DBI does not consider the density-connectivity between points and does not assign points to clusters. Instead, it identifies core points with respect to the *ε* and *minPts* parameters and then calculates a score for each point according to the number of core points in its neighborhood. The points with zero scores are considered outliers and are discarded. The points with scores above a certain threshold are considered core points and are also discarded. The remaining points are considered border points and are retained. The subsequent subsections provide the details of the DBI algorithm.

### 3.1 Identifying core points

In this step, the algorithm identifies core points within the class being analyzed. The algorithm uses similar definitions to DBSCAN. The definitions are provided as follows:

**Definition 3.1 (*ε*-neighborhood)**. The *ε*-neighborhood of a point *p* is defined as:

$$\begin{array}{c} N_{\varepsilon }\left(p\right)=\left\{q\in D|dist\left(p,q\right)\leq \varepsilon \right\} \end{array}$$ (7)

where *D* is the set of all data points in the class and *dist*(*p*, *q*) is the distance between points *p* and *q*. Various distance metrics, such as Euclidean distance, Manhattan distance, etc., may be used for the computation of the distance between two points. This study employs Euclidean distance.

**Definition 3.2 (core point)**. A point whose *ε*-neighborhood contains at least *minPts* neighbors. Formally, a point *p* is considered a core point if:

$$\begin{array}{c} p\in \{q\in D||N_{\varepsilon }\left(q\right)|\geq minPts\} \end{array}$$ (8)

where |*N*_*ε*_(*q*)| is the number of points in the *ε*-neighborhood of *q*.

For every point in the class being analyzed, the *ε*-neighbors count is determined, and core points are identified and marked according to the preceding definition. This step classifies all points in the class being analyzed as either core points or non-core points.

### 3.2 Calculating core scores

In this step, the algorithm calculates a score for each point based on the number of core points in its *ε*-neighborhood. The scores are calculated according to the following definitions:

**Definition 3.3 (core neighbors)**. The core neighbors of a point *p* are the points in the *ε*-neighborhood of *p* that are also core points. The formal definition is as follows:

$$\begin{array}{c} N_{\varepsilon }^{core}\left(p\right)=\{q\in N_{\varepsilon }\left(p\right)||N_{\varepsilon }\left(q\right)\left|\geq minPts\right\} \end{array}$$ (9)

where *N*_*ε*_(*p*) is the *ε*-neighborhood of *p* and |*N*_*ε*_(*q*)| is the number of points in the *ε*-neighborhood of *q*.

**Definition 3.4 (core score)**. The core score of a point *p* is defined as:

$$\begin{array}{c} S\left(p\right)=|N_{\varepsilon }^{core}\left(p\right)| \end{array}$$ (10)

Where $|N_{\varepsilon }^{core}\left(p\right)|$ is the number of core neighbors of *p*.

At this step, each point in the class being analyzed has a core score. These scores can be seen as a measure of how close a point is to the center of the class. The higher the score, the closer the point is to the center of the class. The lower the score, the further the point is from the center of the class. As with most density-based clustering algorithms, DBI assumes that the classes are densest at their centers.

### 3.3 Identifying border points

In this step, points are classified as either border points or outliers. The points with core scores of 0 are considered outliers and are marked as such to be later discarded. For the rest of the points, a threshold *t* is used to decide whether a point is a border point.

Following are the definitions of border outliers and border points.

**Definition 3.5 (outlier)**. An outlier *p* is a point that has a core score of 0. In other words, it has no core neighbors. A formal definition of an outlier point *p* is as follows:

$$\begin{array}{c} p\in \{q\in D|S(q)=0\} \end{array}$$ (11)

**Definition 3.6 (border point)**. A point *p* that has a core score between 1 and a certain threshold *t*. It is formally presented as follows:

$$\begin{array}{c} p\in \{q\in D|1\leq S(q)\leq t\} \end{array}$$ (12)

The threshold *t* is calculated based on a user input ratio *r* ∈ [0, 1], which represents the desired ratio of the size of the reduced dataset to that of the entire training set. *t* is derived by converting the ratio *r* to a quantile of the core scores after removing the outliers. To account for the discarded outliers, an adjusted ratio *q* is calculated as follows:

$$\begin{array}{c} q=\frac{r}{1-\frac{\left|O\right|}{\left|D\right|}} \end{array}$$ (13)

where |*D*| is the size of the class being analyzed and |*O*| represents the number of outliers. The threshold *t* is then calculated as depicted in the following equation:

$$\begin{array}{c} t=Q(\{S(p)|p\in D\backslash O\},q) \end{array}$$ (14)

where *Q* is the quantile function, *S* is the core score function, and O is the set of outliers.

### 3.4 Reducing the training set

In this step, the algorithm discards the outliers and non-border points and retains the border points. The retained border points of all classes are combined to form the reduced training dataset. This dataset is then used to train an SVM classifier.

**Fig 3:**
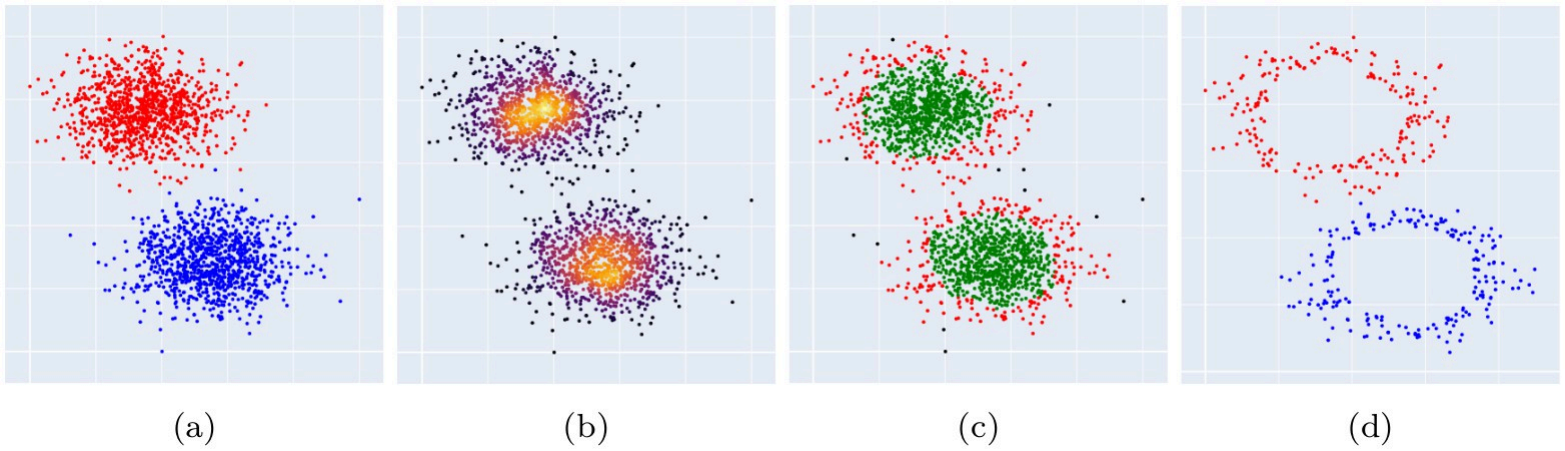
Identification of a layer of border points in our proposed method (DBI). (a) The input classes of a synthetic 2-dimensional dataset set. (b) The calculated core scores for all points of the dataset. (c) The three identified types of points; i.e. border points (red), core points (green) and outliers (black). (d) The final reduced training set.

A summary of the DBI algorithm is presented in Algorithm 1. The main steps of the algorithm are illustrated in Fig 3.

**Algorithm 1** DBI Algorithm

1: **Input:** Dataset *D*, radius *ε*, minimum number of points *minPts*, ratio *r*

2: **Output:** Reduced training set *R*

3: *R* ← ∅

4: **for** each class *C* ∈ {*C*_1_, …, *C*_*n*_}

5:  *O* ← {*q* ∈ *C*∣*S*(*q*) = 0}

6:  $q\leftarrow \frac{r}{1-\frac{\left|O\right|}{\left|C\right|}}$

7:  *t* ← *Q*({*S*(*p*)∣*p* ∈ *C*\*O*}, *q*)

8:  *R* ← *R*∪{*p* ∈ *C*∣1 ≤ *S*(*p*)≤*t*}

9: **end for**

It is relevant to mention that one advantage of DBI is that it considers all borders of classes when selecting border points. This is expected to be useful, especially in multi-class classification problems where most of the border samples contribute to the decision boundaries. This can be seen, for instance, when using dimensionality reduction techniques such as t-SNE (t-Distributed Stochastic Neighbor Embedding) [24], in which local structure is more preserved than global structure, making distances between classes less reliable [25]. Furthermore, embeddings obtained by t-SNE for multi-class datasets tend to have a layout of classes that packs classes close to each other. This layout makes classes have most of their borders adjacent to those of other classes and hence contribute to the decision boundaries. Another advantage is that each of the classes is analyzed independently, which allows for the identification of outliers according to the distribution of each class. This also enables applying techniques to determine suitable parameters specific to each class, such as when classes have different densities. For this study, the same parameters were used for all classes. However, this approach can be easily extended to allow for different parameters for each class. Additionally, DBI enables more control over the number of retained samples by using the user-defined ratio parameter.

### 3.5 Training the SVM classifier

After the reduced training set is obtained, it is utilized to train the SVM classifier. Given that the reduced data set will have a different data distribution than that of the whole dataset, and even though the selected subset is intended to, most likely, contain candidates for the SVs, it is almost impossible to precisely select the exact SVs. This implies that the hyperparameters will deviate from the optimal values that would be obtained by training on the whole dataset. Therefore, the hyperparameters of the SVM classifier must be specifically tuned for optimal performance [7]. In this study, the hyperparameters are tuned using a grid search approach with k-fold cross-validation.

When implementing grid search, it is crucial to ensure that the validation set used to tune the hyperparameters is representative of the target distribution when cross-validation is employed. It should therefore be sampled from the original training dataset. The k-fold technique ensures no overlap occurs between the training and validation sets for every fold, as long as it performs the splitting on a single dataset. However, its implementation is not straightforward in this study since the training fold must be derived from the extracted reduced set. We present a modified implementation of k-fold cross-validation to address this issue. The steps involved in this strategy are as follows:

1. K-fold cross-validation is performed on the original full training set to obtain *k* training and validation folds, *T*_1_, *V*_1_, …, *T*_*k*_, *V*_*k*_.
2. For each fold *i*, a training set $T_{i}^{\prime }$ is constructed by selecting the data points in the reduced set *R* that are also in *T*_*i*_.
3. *T*_*i*_ is discarded while *V*_*i*_ is retained unchanged.
4. The SVM classifier is trained on $T_{i}^{\prime }$ using a set of hyperparameters and validated against *V*_*i*_.
5. The optimal hyperparameters are determined according to the highest mean accuracy across all folds.
6. The SVM classifier is trained on the entire reduced set *R* using the selected hyperparameters.

Notably, because the k-fold split method randomly selects samples for both the training and validation splits, the training subsets from the reduced dataset will have the same ratio to the reduced set as the original training subsets had to the original training set. An advantage of this method is that it permits the extraction of the reduced subset only once prior to grid search or cross-validation, as opposed to applying the extraction step to every training split, which would result in the loss of information provided by the validation subset that was set aside. In addition, the extraction method’s outputs within the k-fold splits may differ significantly from the final output used for training the SVM classifier after grid search, particularly for low values of the number of folds. We argue that our methodology is less sensitive to changes in fold count.

### 3.6 Higher-dimensional datasets

The DBI algorithm is designed to operate on datasets of relatively low dimensionality since its performance is anticipated to degrade as the dataset’s dimensionality increases. This is because the distance between points becomes less reliable, which is a characteristic of the *curse of dimensionality* [26]. To circumvent this limitation, we suggest employing the UMAP (Uniform Manifold Approximation and Projection) dimensionality reduction technique [27]. It is a method of non-linear dimensionality reduction based on manifold learning. It is intended to conserve the data’s local structure, to be computationally efficient, and to scale well to large datasets. In our method, UMAP is intended to be used to transform the dataset to lower dimensions, such as 2 or 3 dimensions, before applying the data selection phase of DBI. Once the reduced dataset is identified, it can be mapped to any higher-dimensional embeddings for the training phase using the indices of the data points in the original dataset.

The use of UMAP for enhancing DBSCAN performance was investigated in [28]. The authors argued that the use of UMAP improves the performance of the DBSCAN algorithm by reducing its parameter sensitivity. We argue that reducing the dimensionality of the dataset for data reduction will be beneficial for allowing repetitive training of the SVM classifier more efficiently. This is usually needed for model selection, hyperparameter tuning, and cross-validation. We also argue that the use of UMAP for dimensionality reduction will be specifically favorable in this case for the following reasons:

- The embeddings that are produced by UMAP maintain the data’s local structure, which is expected to be useful for DBI, which relies on local structure.
- UMAP tends to produce more compact clusters in their embeddings compared to other dimensionality reduction techniques such as t-SNE [29].
- Given its topological assumptions about the distribution of points on the lower-dimensional manifold, UMAP tends to favor a certain degree of uniformity of cluster density [27].
- It was observed during experimentation that most of the support vectors identified in higher-dimensional embeddings tend to reside at the boundaries of the compact clusters in the lower-dimensional embeddings. This is assumed to make the extraction of a border layer in lower-dimensional embeddings more relevant.
- It was also observed that in higher-dimensional embeddings produced by UMAP, the number of resulting support vectors is significantly lower than that of the original dimensionality. This is expected to result in a faster prediction time. This contrasts with the higher number of support vectors detected in higher-dimensional embeddings produced by linear dimensionality reduction techniques such as PCA (Principal Components Analysis).

### 3.7 Variants of the DBI algorithm

In DBI, we focused on the extraction of a layer of border points to represent the reduced set; however, this method is sensitive to the distribution of the dataset, and therefore the performance of the algorithm is expected to vary depending on the dataset. One limitation was observed in cases of datasets with significant overlap between their classes. In such cases, excessive reduction of the dataset will result in very thin layers of border points. Such overlapping border layers will represent a considerable ratio of the selected subset, and therefore instances from different classes crossing the decision boundary will interfere with the construction of a suitable decision boundary and result in a degraded performance of the final classifier. Another drawback is that with overlapping borders, most of the data points in the overlapping area will be considered support vectors. This results in a high number of support vectors and, hence, a slower prediction time. In response to these constraints, we introduce the following variations to the DBI algorithm:

#### 3.7.1 BRI (Border-biased Random Instance selection)

In this variant, DBI is extended to combine two scoring schemes: the *core score* and a *pureness score*. The core score is the same as the core score used in DBI, with an additional normalization step to range from 0 to 1. The pureness score is calculated for a point as the ratio of the number of *k* nearest neighbors sharing the same class label to the total number of *k* nearest neighbors. The pureness score is then added to the core score to obtain the final score of the point. Outliers will have a core score of 0 and will be discarded. It is worth noting that the final score will be higher for points that are further away from the borders of classes and those that are not in overlapping areas. The score is then used as the weight for a weighted random sampling of points to be discarded from the reduced training set. This method shares the same parameters as DBI with the additional *k* parameter. The *k* parameter is used to control the number of neighbors used to calculate the pureness score. The pureness score is calculated as follows:

$$\begin{array}{c} pureness\left(p\right)=\frac{\left|\{q\in N_{k}\left(p\right)|q\in C\}\right|}{|N_{k}\left(p\right)|} \end{array}$$ (15)

where *N*_*k*_(*p*) is the *k* nearest neighbors of *p* and *C* is the class of *p*. The final score is calculated as follows:

$$\begin{array}{c} score\left(p\right)=\{\begin{array}{cc} S^{\prime }\left(p\right)+pureness\left(p\right) & \text{if}\;S(p)\neq 0\\ 0 & \text{otherwise} \end{array} \end{array}$$ (16)

where *S*^′^(*p*) is the normalized core score of *p* which is calculated as follows:

$$\begin{array}{c} S^{\prime }\left(p\right)=\frac{S\left(p\right)-\min_{q\in C}S(q)}{\max_{q\in C}S(q)-\min_{q\in C}S(q)} \end{array}$$ (17)

This variant, in other words, randomly selects points for removal from the dataset with a high probability for points that are more central and that have pure neighborhoods. This is equivalent to randomly selecting points for retention with a high probability for points that are closer to the borders of classes and that lie in overlapping areas. Therefore, it aims to overcome the first limitation.

#### 3.7.2 BRIX (border-biased random instance selection with eXclusion)

To address the second limitation, we propose a variant that aims to reduce the number of detected SVs. In this variant, the same scoring scheme used in BRI is used to calculate the score of each point. However, instead of using the pureness score directly, its complement is used as a measure of how impure the neighborhood of the point is. The final score is calculated as follows:

$$\begin{array}{c} score\left(p\right)=\{\begin{array}{cc} S^{\prime }\left(p\right)+\left(1-pureness\left(p\right)\right) & \text{if}\;S(p)\neq 0\\ 0 & \text{otherwise} \end{array} \end{array}$$ (18)

**Fig 4:**
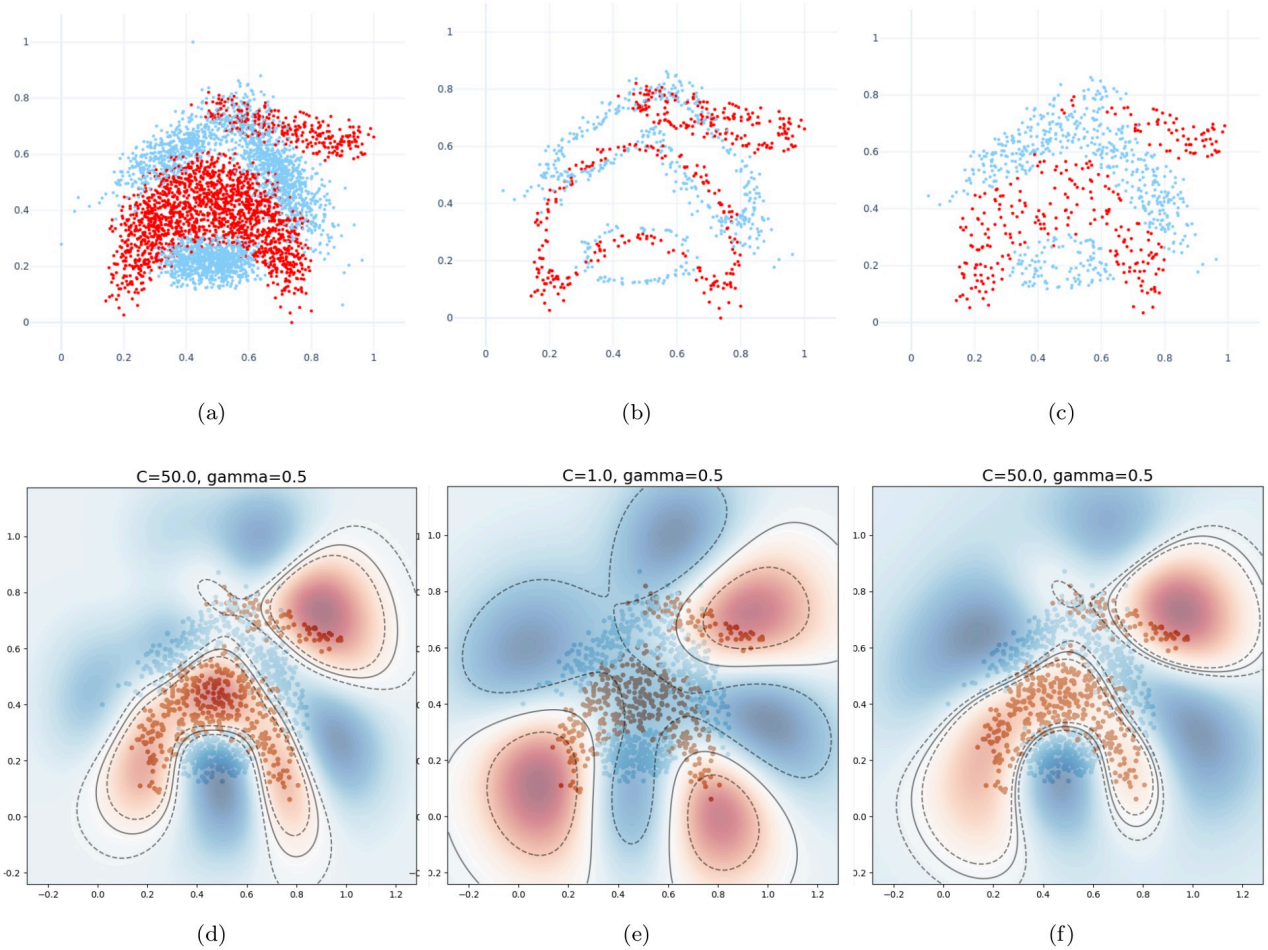
Effect of border overlap on the decision boundaries of the trained classifier on the Banana dataset. The whole dataset is compared to our proposed DBI and BRIX methods, both at a ratio of 0.2. The test dataset is plotted against the decision boundaries (solid lines) and margins (dashed lines). (a) Whole dataset. (b) Reduced dataset (DBI). (c) Reduced dataset (BRIX variant). (d) Test dataset plot against decision boundaries (whole dataset). (e) Test dataset plot against decision boundaries (DBI). (f) Test dataset plot against decision boundaries (BRIX variant). Notice the misclassified points in the central region of the plot in (e) due to the overlapping borders of the opposite class around the central part.

The remaining steps are identical to the BRI variant. This variant thus randomly selects points for removal, with a high probability for points that are more central and that have impure neighborhoods. This is equivalent to randomly selecting points for retention with a high probability for points that are closer to the borders of classes and that lie away from overlapping areas. As a result, the reduced dataset will have less overlap between classes, as can be seen in Fig 4.

#### 3.7.3 SVO (Support Vector Oracle)

**Fig 5:**
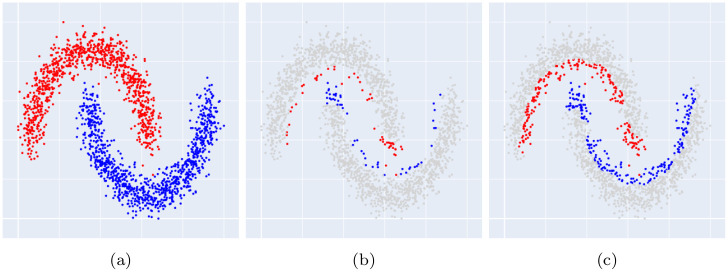
Reduced dataset selection using the proposed SVO method for a non-overlapping dataset. (a) A moons-shaped dataset. (b) Identified support vectors. (c) Reduced subset using SVO at *k* = 15.

Here we propose a different approach to border layer extraction that is independent of the DBI density-based approach. The Support Vector Oracle (SVO) is intended for use with datasets of higher dimensions. It identifies support vectors in lower-dimensional embeddings produced by UMAP by training an SVM classifier. Then it uses the identified support vectors as seeds to construct the reduced dataset by selecting the nearest neighbors sharing the same label. Therefore, it requires the parameter *k* which defines the number of nearest neighbors to collect. An example is illustrated in Fig 5. The constructed reduced dataset is then used for training SVM classifiers after mapping its data points to the original dataset based on their original indices. They can also be mapped to other higher-dimensional UMAP embeddings of the original dataset.

#### 3.7.4 SVOX (Support Vector Oracle with eXclusion)

This is a variant of the SVO that aims at a smaller number of identified support vectors using a similar strategy to the BRIX variant. Specifically, it excludes support vectors with impure neighborhoods from the seeds of identified support vectors according to a defined threshold. The remaining steps are similar to the SVO variant, except that only data points with pure neighborhoods are considered neighbors. The support vectors to be included as seeds are selected as follows:

$$\begin{array}{c} SV\left(p\right)=\{\begin{array}{cc} p & \text{if}\;pureness(p)\geq L\\ \text{None} & \text{otherwise} \end{array} \end{array}$$ (19)

where *pureness*(*p*) is the neighborhood pureness measure for *p* as defined in Eq 15, and *L* is a user-defined threshold. Similar to SVO, *k* controls the extent of the neighbors to be collected. Another variable, *N* manipulates the number of neighbors to consider when estimating the pureness measure. The excluded support vectors and their neighbors are more likely to be found in areas of overlap between classes. This variant is thus expected to have an advantage in such cases.

## 4 Experiments

This section details the experimental setup and the outcomes of applying the DBI method and its variants to different datasets.

### 4.1 Datasets

The DBI algorithm and its variants were assessed on synthetic and real-world datasets. The datasets used are briefly described in this section.

#### Banana

A two-dimensional dataset that consists of two classes that show considerable overlap. It consists of 5,300 samples. In our experiments, the training dataset contains 4,240 samples, while the testing dataset has 1,060 samples [30].

#### USPS

This dataset consists of 10 classes of handwritten digits. 7,291 samples are provided for training and 2,007 for testing. Every sample is a 16x16 grayscale image automatically scanned from an envelope by the U.S. Postal Service [31].

#### Adult9a

One of the categories included in the Adult dataset. It comprises 32,561 and 16,281 samples for training and testing, respectively. Every instance represents an individual with 123 characteristics. The objective of this binary classification problem is to predict whether an individual earns more than $50,000 annually. With 24,720 samples in the negative class and 7,841 samples in the positive class, the dataset is extremely unbalanced. The dataset is preprocessed by eliminating samples with missing values and encoding categorical attributes with one-hot encoding [32].

### 4.2 Metrics

The predictive performance of the classifier trained by employing the DBI algorithm and its variants is assessed using the classification *accuracy* as defined in Eq 20.

$$\begin{array}{c} accuracy=\frac{TP+TN}{TP+TN+FP+FN} \end{array}$$ (20)

where *TP* denotes true positives, *TN* represents true negatives, *FP* is false positives, and *FN*, false negatives. Equivalently, it represents the proportion of accurate predictions out of all predictions made.

For the experiments, the accuracy is evaluated against the testing dataset, which is not used during the training phase and hence is not considered during the instance selection process. A baseline accuracy is calculated by training the SVM classifier on the entire training dataset and evaluating its accuracy. This baseline score is then contrasted with the accuracy of a model trained only on the reduced portion of the data. Another metric that is used as a potential indicator of the quality of the selected training subset is the *number of support vectors* identified by the SVM classifier. Minimizing this number will speed up the prediction time of the SVM classifier and reduce its memory requirements. An interesting metric that is used for exploratory analysis is a measure of how similar the support vectors identified in the selected training subset are to those identified in the full training dataset. For this purpose, the *Jaccard similarity coefficient* is used. It is described in Eq 21 for two sets *A* and *B*, as the proportion of the intersection of the two sets to their union [33].

$$\begin{array}{c} J\left(A,B\right)=\frac{\left|A\cap B\right|}{\left|A\cup B\right|} \end{array}$$ (21)

*Training time* and *testing time* are also measured for both the baseline classifier and that trained on the reduced subset. The training time is measured as the time the SVM classifier takes to train once using the tuned hyperparameters identified by grid search for each of the compared classifiers. The testing time is measured as the time it takes to predict the labels of the test set using the trained SVM classifier.*Training speedup* and *testing speedup* are calculated as the ratio of the training time of the baseline classifier to that of the reduced subset and the ratio of the testing time of the baseline classifier to that of the reduced subset, respectively.

### 4.3 Experimental setup

The experiments were conducted using Python 3.8.10 on a machine with an Intel Core i7–10750H CPU, an NVIDIA GeForce GTX 1660 Ti GPU, and 16 GB of RAM running Ubuntu 20.04.1 LTS. The experiments were conducted using the scikit-learn library [34]. The SVM classifier was trained using the SVC class and the RBF as the kernel. The regularization hyperparameter C was tuned separately for each of the compared classifiers using a grid search approach. It was selected from the values (1, 10, and 50). The kernel coefficient *γ* was set to the default value calculated by the SVC class, which is estimated as follows:

$$\begin{array}{c} \gamma =\frac{1}{d\times Var(X)} \end{array}$$ (22)

where *X* is the training data, *d* is the number of its attributes, and *Var*(*X*) is its variance. Therefore, since the training data is standardized before training, i.e., scaled to unit variance, it will be 1/*d*. A different set of the SVM hyperparameters was tuned for the Adult9a dataset, where C was selected from the values (128, 256, and 512) and *γ* was selected from the values (5 × 10^−5^, 1 × 10^−4^, 5 × 10^−4^). The grid search was performed using 5-fold cross-validation for the classifier training on the whole dataset, and a customized 5-fold cross-validation, as described in Section 3.5, was employed for training on the reduced dataset. For DBI and its variants, *minPts* was fixed at 6, while *ε* was fixed at 0.5 for both USPS and Adult9a datasets and 0.05 for the Banana dataset for all experiments. For BRI and BRIX, *k* was set to 15. All the experiments were repeated 10 times with different random seeds, where appropriate. The outcomes presented are the mean over the 10 runs.

For the multidimensional datasets, feature extraction and dimensionality reduction were applied using UMAP [27] with the default parameters. The USPS dataset was reduced to 64 dimensions for training the SVM classifier, and it was reduced to 2 and 3 dimensions for applying the data reduction using DBI or its variants. An exception to this is the Adult9a dataset, which is only reduced to 2 dimensions for border layer extraction while training occurs on the original dimensionality, which has a relatively low number of features (123).

### 4.4 Comparison methodology

In our study, a comparative approach was adopted to evaluate the performance of our proposed methods against other existing methods. This comparison was not solely based on a single attribute, such as accuracy, but rather considered multiple objectives simultaneously. This approach aligns with the multi-objective nature of the problem of training data reduction for SVM models, which involves balancing training speedup, testing speedup, accuracy, and memory requirements (e.g., the number of SVs). Balancing these objectives presents a significant challenge, as improving one aspect may lead to the degradation of another. Therefore, careful consideration of trade-offs among these objectives is essential for identifying the optimal solution for a given application.

Our adopted approach involved selecting the best point for each method such that it achieved the maximum reduction of the data while maintaining an accuracy not lower than a certain threshold from the original accuracy of the model trained on the entire dataset (i.e., the baseline classifier). This approach allowed for the identification of solutions that offered significant data reduction without compromising too much on accuracy.

Additionally, we employed the concept of the Pareto set to further refine our analysis in a similar approach to that used in [16]. The *Pareto set*, in the context of multi-objective optimization, represents a set of solutions that are considered optimal in the sense that no other solutions in the search space are superior to them when all objectives are considered. These solutions are also known as *non-dominated*, Pareto optimal, or efficient solutions. A solution is said to dominate another solution if it is better or equal in all objectives and strictly better in at least one objective. In other words, a solution is dominated if there exists another solution that improves at least one objective without worsening the others. In that sense, each solution in the Pareto set offers a unique trade-off among the multiple objectives [35].

For identifying the Pareto set, we consider the following metrics as possible objectives to optimize:

- *Error rate*: This metric quantifies the proportion of incorrect predictions made by the model. It is computed as (1−accuracy), where accuracy is as defined in Eq 20.
- *Training time ratio*: This metric is the ratio of the training time on the reduced subset to that on the entire dataset (i.e., 1/training speedup). A lower training time ratio is indicative of a more computationally efficient model, as it denotes a reduction in the time required for training when utilizing the reduced dataset.
- *Testing time ratio*: Analogous to the training time ratio, this metric is the ratio of the testing time on the reduced subset to that on the entire dataset (i.e., 1/testing speedup). A lower testing time ratio is preferable, as it signifies an enhancement in prediction speed when employing the reduced dataset.

The elements of the Pareto set were ranked based on their Euclidean distance from the optimal point, which provided valuable insights into the best points in terms of the trade-off between speedup and accuracy.

The score, representing closeness to the optimal point, is calculated as follows:

$$\begin{array}{c} score=\frac{1}{\sqrt{\sum_{i=1}^{n}{\left(x_{i}-x_{i}^{*}\right)}^{2}}} \end{array}$$ (23)

where *x*_*i*_ is the value of the *i*-th objective for the solution under consideration, $x_{i}^{*}$ is the value of the *i*-th objective for the optimal point, and *n* is the number of objectives. The optimal point in this context would be the zero point, representing the ideal of minimizing all the optimized metrics.

An interesting alternative for distance calculation is the weighted Euclidean distance, which allows for the incorporation of weights into the different objectives to reflect their relative importance for the target application. However, the Euclidean distance was used in this study, as it is more intuitive and straightforward to interpret. This multi-objective optimization perspective enabled a more comprehensive and nuanced evaluation of the methods under consideration.

### 4.5 Results

The findings of applying the DBI method and its variants to the various datasets are presented in this section.

#### 4.5.1 Banana

**Fig 6:**
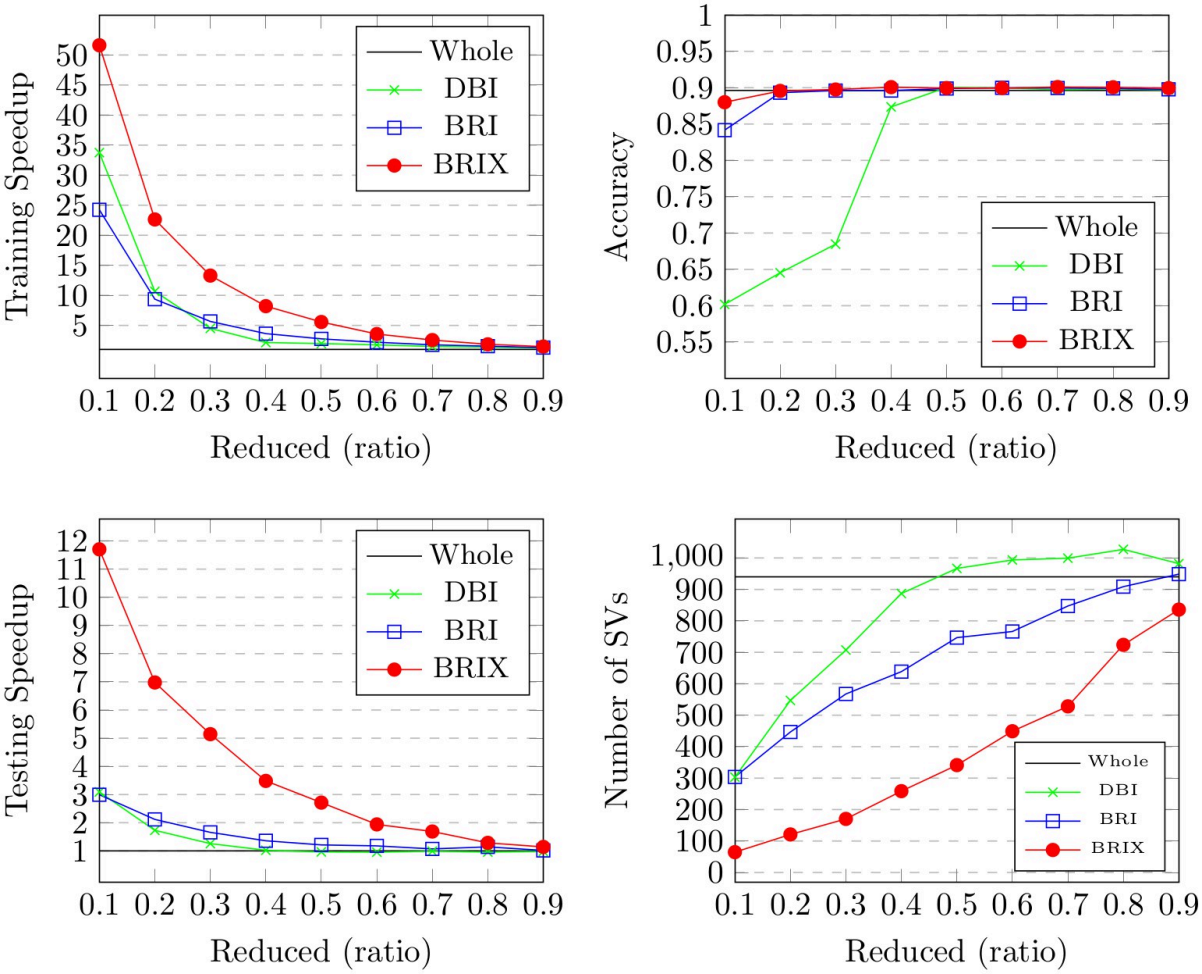
Results for our proposed methods (DBI, BRI and BRIX) on the Banana dataset with different reduction ratios.

For the Banana dataset, different target ratios for the reduced dataset were verified. The outcomes are detailed in Table 1 and Fig 6. BRIX shows an accuracy of 0.88 at a ratio of 0.1, which is the highest accuracy compared to the other variants at the same ratio. BRIX and BRI show similar accuracies around 0.9 for ratios 0.2 and higher, which are on par with those of the whole dataset. There is a relatively lower accuracy for DBI, with a sharp rise in accuracy from 0.68 to 0.87 at a ratio of 0.4. This can be due to the observation that the reduced dataset obtained using DBI with ratios below this value has a significantly different distribution due to its relatively thin border layers, as shown in Fig 4. These border layers overlap, crossing to the opposite side of the expected decision boundary. This confuses the grid search during model selection. For example, at ratio 0.2, the selected regularization parameter C value was 1, which is considerably lower than that of 50, which was selected at ratio 0.4. This results in misidentified decision boundaries and relatively degraded accuracy below this ratio. Ratios of 0.4 and above, on the other hand, show increasingly improved decision boundaries closer to those identified by training on the whole dataset, which can be attributed to having enough training data points to help the grid search find a suitable regularization parameter. BRIX also achieves the highest training speedup, starting at 51 for the ratio of 0.1, with a sharp decline reaching around 13 at the ratio of 0.3, after which the decline slows down. DBI and BRI have similar declines but with earlier sharp drops and lower starting values of around 35 and 25, respectively.

**Table 1:** Results of the proposed methods (DBI, BRI & BRIX) on the Banana dataset with different reduction ratios.

Ratio	Training Speedup			Testing Speedup			Accuracy			Number of SVs			Jaccard similarity		
Whole dataset	1.00			1.00			0.896			940			1.00		
	DBI	BRI	BRIX	DBI	BRI	BRIX	DBI	BRI	BRIX	DBI	BRI	BRIX	DBI	BRI	BRIX
0.1	33.75	24.24	51.62	3.09	2.99	11.70	0.602	0.842	0.880	303	304	65	0.19	0.23	0.05
0.2	10.66	9.36	22.63	1.73	2.12	6.98	0.645	0.893	0.896	547	447	121	0.36	0.34	0.11
0.3	4.50	5.66	13.29	1.26	1.66	5.14	0.685	0.896	0.897	707	568	170	0.53	0.43	0.16
0.4	2.15	3.63	8.20	1.02	1.36	3.48	0.874	0.896	0.901	887	639	259	0.78	0.52	0.25
0.5	1.98	2.76	5.57	0.96	1.21	2.71	0.900	0.899	0.900	967	747	341	0.88	0.57	0.33
0.6	1.76	2.20	3.56	0.96	1.18	1.94	0.900	0.900	0.900	994	766	449	0.90	0.67	0.42
0.7	1.50	1.77	2.56	0.99	1.07	1.69	0.897	0.900	0.901	999	847	528	0.92	0.73	0.54
0.8	1.39	1.55	1.85	0.96	1.14	1.28	0.898	0.899	0.901	1027	909	724	0.89	0.79	0.65
0.9	1.22	1.31	1.47	0.98	1.02	1.14	0.897	0.898	0.899	982	949	836	0.94	0.86	0.80

Each row compares performance metrics of the proposed methods for a different value (0.1 to 0.9) of the reduction ratio parameter *r* (the user-defined desired proportion of the training set to retain). The first row represents the metrics measured after training on the whole dataset.

**Fig 7:**
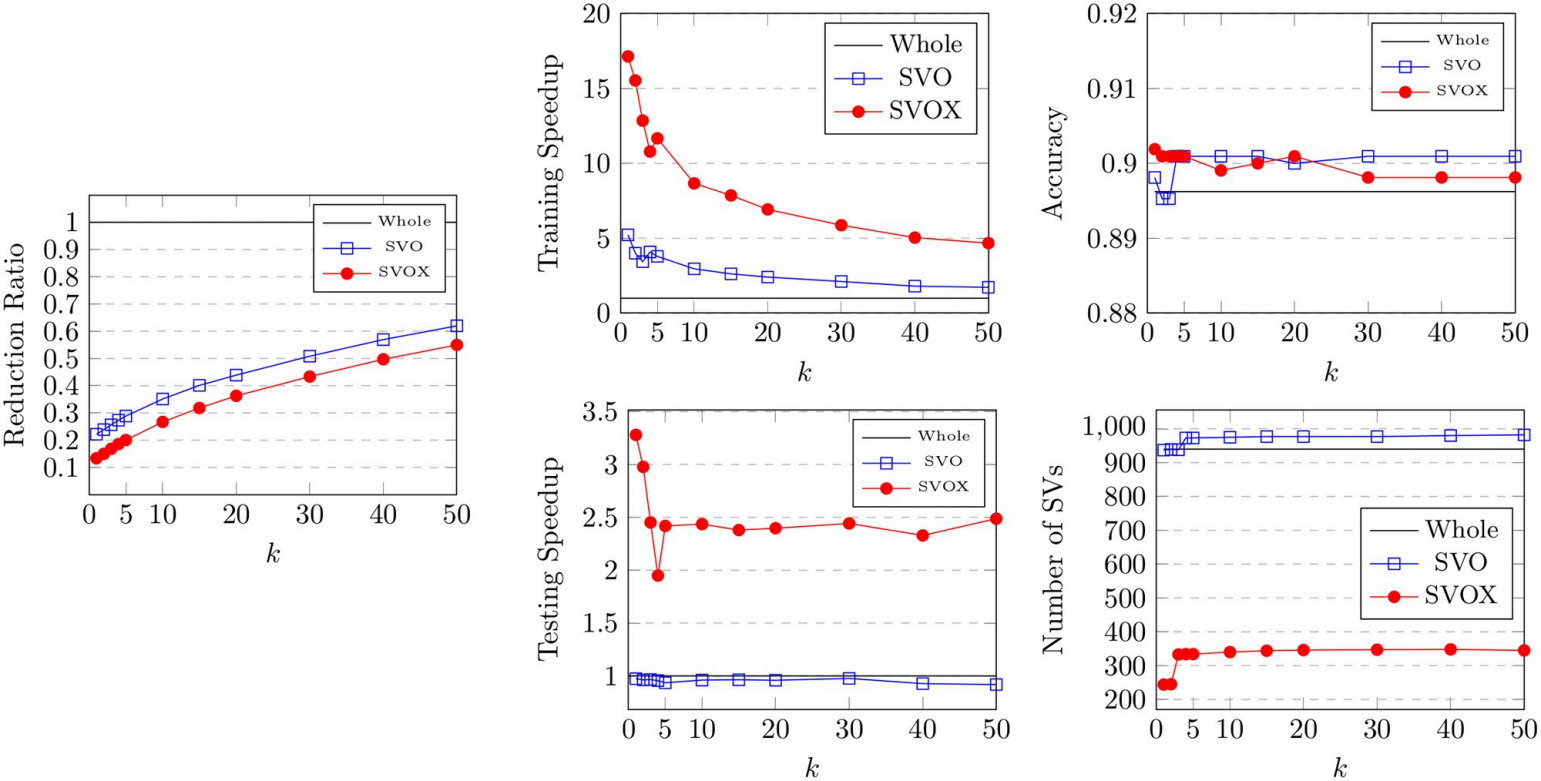
Results for the proposed SVO and SVOX methods on the Banana dataset with different values of *k*.

The BRIX variant also achieves a significant speedup in classifier testing time. This reaches 12 at the lowest ratio of 0.1 and slowly declines for higher ratios. On the other hand, very minimal testing speedup is obtained by BRI and DBI. This is attributed to the substantial reduction in support vectors obtained by BRIX, particularly for low reduced dataset ratios. This can, in turn, be attributed to the fact that BRIX favors selection from non-overlapping regions in contrast to the other variants. It is evident in Fig 4 that the reduced set selected by DBI shows a significant overlap in the border layers of the two classes, while the overlap is much reduced for the BRIX variant. For completeness, SVO and SVOX were also tested against the Banana dataset for different values of *k*. The results are presented in Fig 7 and Table 2. SVO and SVOX maintained nearly stable accuracy across all tested values of *k*. However, the training speedup for SVOX was about 3 times that obtained by SVO. The testing speedup was highest at *k* = 1 for SVOX at a value of 3.28 and declines rapidly as *k* reaches 5, after which it is nearly constant around 2.5. SVO shows slightly slower prediction times than the baseline classifier for all values of *k*. This is attributed to the fact that the number of support vectors identified by SVO slightly exceeds that of the baseline classifier. These findings suggest that a possible use case for SVOX when applied to low-dimensional datasets, at their original dimensionality, is to reduce the size of the trained model through the reduction of the number of support vectors, resulting in a faster prediction time.

**Table 2:** Results of the proposed SVO & SVOX methods on the Banana dataset with different values of *k*.

*k*		Reduced (ratio)		Training Speedup		Testing Speedup		Accuracy		Number of SVs		Jaccard similarity	
Whole dataset		1.00		1.00		1.00		0.896		940		1.00	
	SVO	SVOX	SVO	SVOX	SVO	SVOX	SVO	SVOX	SVO	SVOX	SVO	SVOX
1	0.22	0.13	5.23	17.14	0.97	3.28	0.898	0.902	937	244	1.00	0.26
2	0.24	0.15	4.00	15.53	0.96	2.98	0.895	0.901	939	245	0.98	0.26
3	0.26	0.17	3.44	12.85	0.97	2.45	0.895	0.901	938	333	0.98	0.35
4	0.27	0.19	4.08	10.78	0.96	1.95	0.901	0.901	973	334	0.95	0.35
5	0.29	0.20	3.79	11.67	0.94	2.42	0.901	0.901	973	334	0.95	0.35
10	0.35	0.27	2.96	8.66	0.96	2.44	0.901	0.899	975	340	0.94	0.35
15	0.40	0.32	2.62	7.86	0.96	2.38	0.901	0.900	977	344	0.94	0.36
20	0.44	0.36	2.41	6.92	0.96	2.40	0.900	0.901	977	346	0.94	0.36
30	0.51	0.43	2.11	5.87	0.98	2.44	0.901	0.898	977	347	0.94	0.36
40	0.57	0.50	1.80	5.04	0.93	2.33	0.901	0.898	980	348	0.94	0.36
50	0.62	0.55	1.73	4.67	0.92	2.49	0.901	0.898	982	345	0.94	0.36

Each row compares performance metrics of the methods for a different value (1 to 50) of the parameter *k* of the SVO & SVOX methods. The first row represents the metrics measured after training on the whole dataset. “Reduced (ratio)” is the ratio of the size of the resulting reduced dataset to the size of the whole dataset.

The DBI algorithm and its variants were compared experimentally to the CBCH algorithm [15]. The authors claimed that CBCH, with the number of clusters K = 50, yielded an accuracy of 0.95, but the actual mean accuracy obtained after 10 runs of the experiment was 0.89. Notably, the 0.90 accuracy obtained by training the SVM on the entire dataset is extremely unlikely to be surpassed by any other method without overfitting the data. This is assumed given that there is a substantial overlap between the two dataset classes. Also observed was an average reduced ratio of 0.70 obtained at k = 100. The Jaccard index is high (0.93) primarily because CBCH retains instances in areas of overlap between the two classes, and most of those are considered support vectors by the SVM algorithm. In addition, the proposed methods are compared to FIFDR [13] and BPLSH [16] according to reported results in the literature [17]. The compared results are presented in Table 3. In our comparisons, the records for our proposed methods were selected such that they have the minimum reduced dataset while attaining an accuracy of not less than 0.02 from the baseline classifier. It is observed that the BRIX variant outperforms the compared methods and the other variants in terms of training speedup, testing speedup, reduction ratio, and support vector count at a reduced ratio of 0.1 and an acceptable accuracy of 0.88, which is on par with that of the other methods. SVOX shows the highest accuracy of 0.9 and ranks second after BRIX in terms of the other metrics. DBI, SVO, and CBCH do not achieve any significant speedup in testing time. This is attributed to the number of support vectors identified being nearly equivalent to that of the baseline classifier. It is also noted that CBCH, FIFDR, and DBI require a reduced dataset of more than half of the original dataset to obtain adequate accuracy relative to the baseline classifier.

**Table 3:** Results of the proposed methods compared to other methods from the literature on the Banana dataset.

Method		Reduced (ratio)	Accuracy	Training Speedup	Testing Speedup	Reduced SVs (ratio)
**Whole dataset**		1.00	0.896	1.00	1.00	1.00
**FIFDR [13]**		0.684	0.877	4.99	1.741	0.315
**CBCH (*K* = 100) [15]**		0.70625000	0.899057	1.540797	0.968350	1.024043
**BPLSH (*M* = 90, *L* = 10) [16]**		0.24707547	0.881887	5.138549	1.077517	0.857766
**Proposed Methods**	**DBI (*r* = 0.5)**	0.5	0.900	1.98	0.96	1.028
	**BRI (*r* = 0.2)**	0.2	0.893	9.36	2.12	0.47
	**BRIX (*r* = 0.1)**	**0.1**	0.88	**51.62**	**11.7**	**0.068**
	**SVO (*k* = 1)**	0.2217	0.898	5.23	0.97	0.996
	**SVOX (*k* = 1)**	0.1335	**0.902**	17.14	3.28	0.26

The first row represents the metrics measured after training on the whole dataset. “Reduced (ratio)” is the ratio of the size of the resulting reduced dataset to the size of the whole dataset. “Reduced SVs (ratio)” is the ratio of the number of SVs of the reduced dataset to that of the whole dataset.

**Fig 8:**
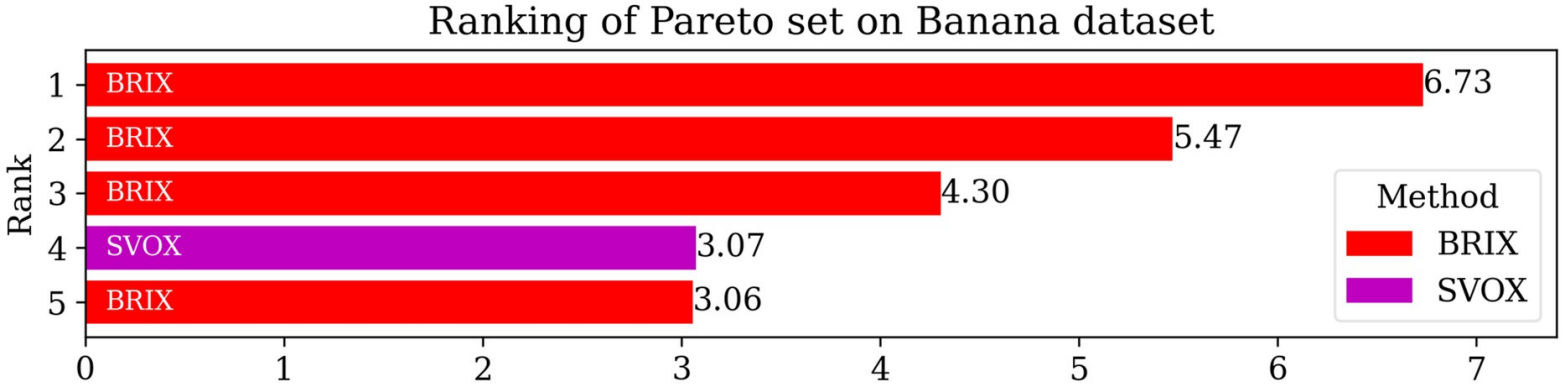
Ranking of Pareto set methods on the Banana dataset based on closeness to the optimal point. The optimal point is the zero point, representing the ideal of minimizing all the optimized metrics. The score is calculated as the reciprocal of the Euclidean distance from the optimal point.

For additional insights into the performance of the proposed methods, the Pareto set for the proposed and compared methods was identified. The objectives considered for the Pareto set are the error rate, training time ratio, and testing time ratio. The Pareto set elements are listed in Table 4. After the elimination of 70 non-dominating solutions, the set is composed of only five elements. These elements are exclusively proposed methods, namely BRIX and SVOX. BRIX constitutes four elements of the Pareto set, while SVOX constitutes only one. The ranking of the Pareto set elements based on closeness to the optimal point is represented in Fig 8.

**Table 4:** Pareto set of different methods on the Banana dataset.

Method	Reduced (ratio)	Accuracy	Training Speedup	Testing Speedup	Reduced SVs (ratio)	Rank
BRIX (*r* = 0.1)	0.10	0.880	51.62	11.70	0.07	1
BRIX (*r* = 0.2)	0.20	0.896	22.63	6.98	0.13	2
BRIX (*r* = 0.3)	0.30	0.897	13.29	5.14	0.18	3
SVOX (*k* = 1)	0.13	0.902	17.14	3.28	0.26	4
BRIX (*r* = 0.4)	0.40	0.901	8.20	3.48	0.28	5

Methods in the Pareto set are ordered by their rank according to closeness to the optimal solution. “Reduced (ratio)” is the ratio of the size of the resulting reduced dataset to the size of the whole dataset. “Reduced SVs (ratio)” is the ratio of the number of SVs of the reduced dataset to that of the whole dataset.

**Fig 9:**
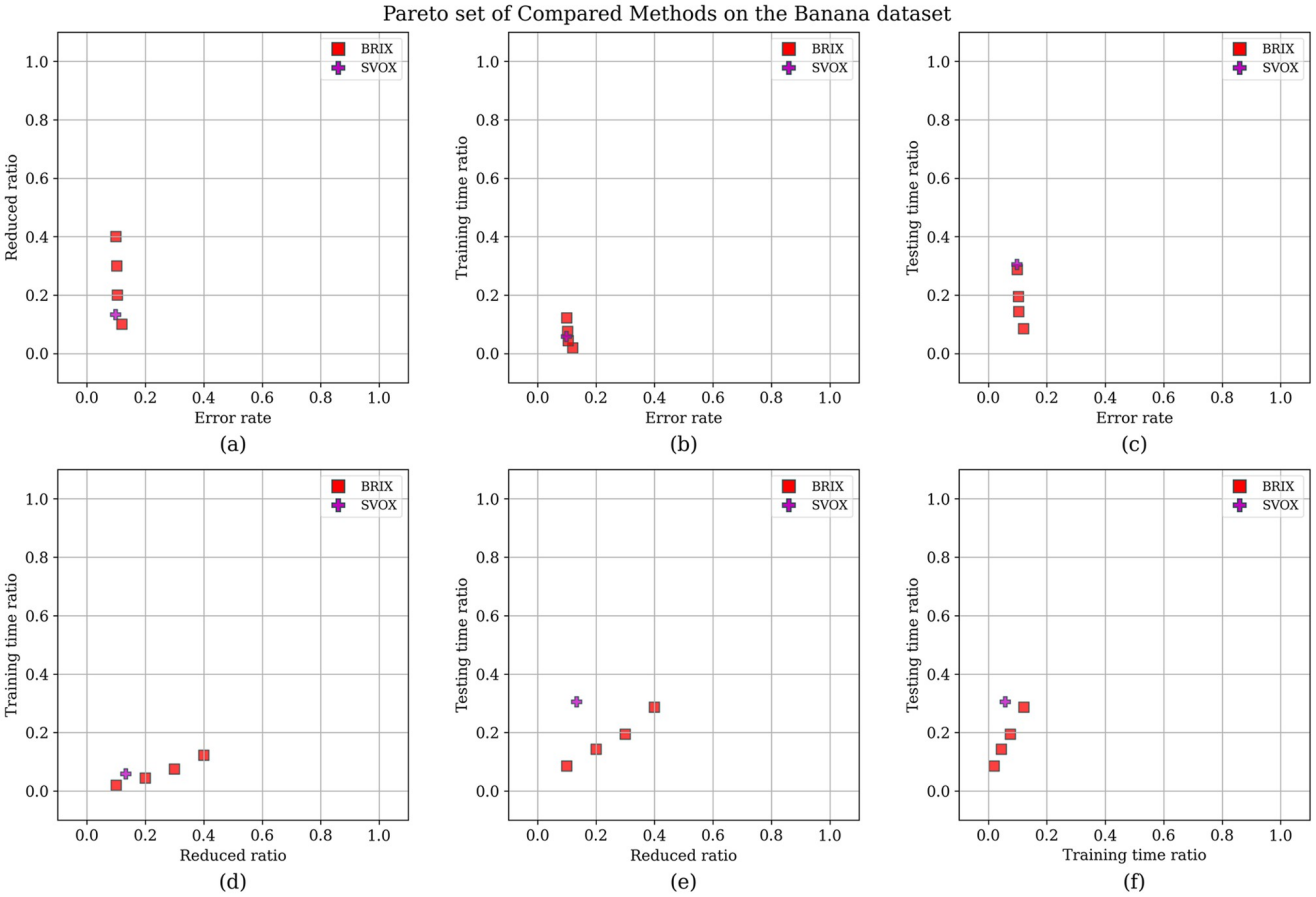
Pareto set for the Banana dataset. After the elimination of 70 non-dominating solutions, the set is composed of only five elements. These elements are exclusively proposed methods, namely BRIX and SVOX. (A point is dominating if it is better or equal in all objectives and strictly better in at least one objective).

**Fig 10:**
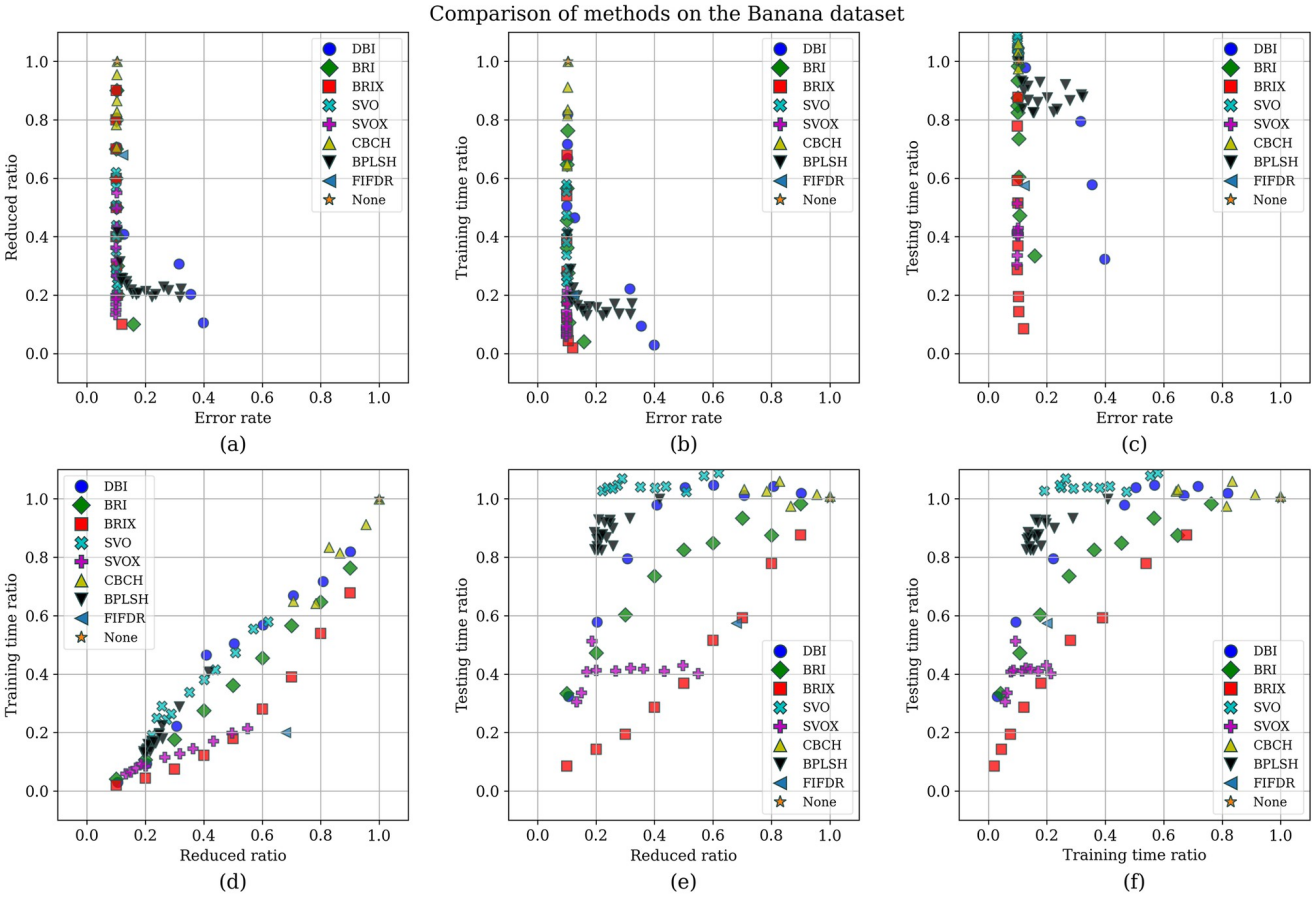
Comparison of methods on the Banana dataset. The distribution of the different methods in the solution space of the three optimization objectives, in addition to the ratio of the reduced dataset, is shown for each pair of objectives. The proposed methods, except DBI and SVO, are predominantly closest to the optimal point.

To enhance the analysis of the Pareto set, the different trade-offs presented by each of the Pareto set elements are visualized in Fig 9. It can be observed that BRIX has closer points to the optimal point than SVOX for all the objectives. Furthermore, the distribution of the different compared methods in the solution space of the three optimization objectives, in addition to the ratio of the reduced dataset, is investigated and presented in Fig 10. The proposed methods, except DBI and SVO, predominate in the set of closest points to the optimal point. It is also evident that the proposed methods are superior in terms of training time ratios and exclusively dominate testing time ratios below 0.5.

#### 4.5.2 USPS

**Fig 11:**
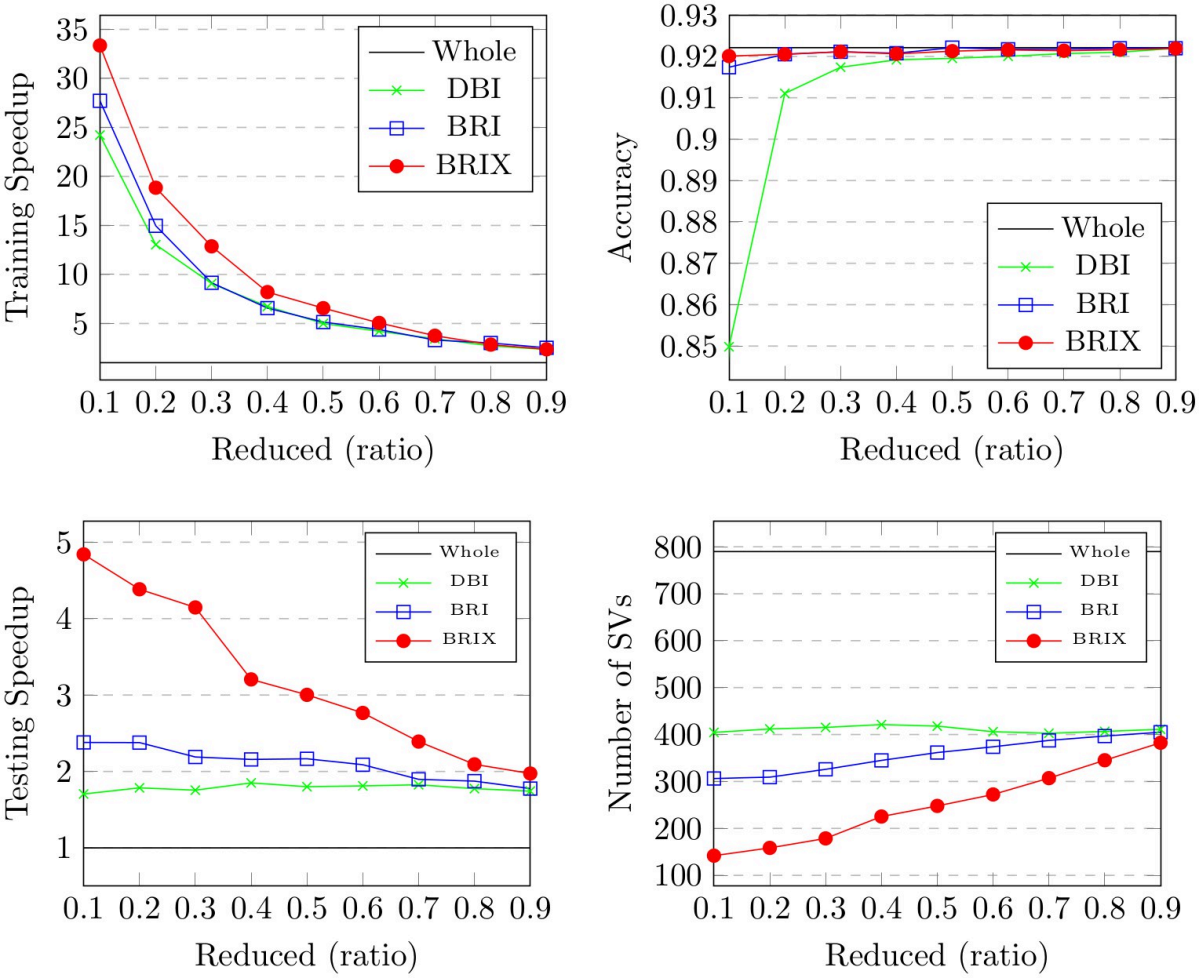
Results for the proposed methods (DBI, BRI and BRIX) on the USPS dataset with different reduction ratios.

For the USPS dataset, we experimented with applying the DBI algorithm to the 2-dimensional UMAP embeddings while training the SVM classifiers on both the whole dataset and the reduced one using our method using 64-dimensional embeddings. The results are presented in Fig 11 and Table 5. The findings exhibit similar patterns to those observed in the Banana dataset regarding the training speedup. However, the testing speedup is much lower for the USPS dataset. DBI demonstrates an almost constant testing speedup of around 2 for ratios 0.1 and above. Interestingly, it also shows a nearly constant value of around 400 for the number of identified support vectors, which is about half the number for the whole dataset. This observation aligns with the linear inverse relationship between speedup and the number of support vectors. It may also be explained by the preserved association between local structures in both 2-dimensional and 64-dimensional UMAP embeddings. BRIX achieved the highest speedup of 62.17 for training and 5.78 for testing, with an acceptable accuracy of 0.915 at a ratio of 0.04. Testing speedup declined almost linearly with increasing the ratio of the reduced set for ratios above 0.05. BRI and BRIX maintained an almost constant accuracy of 0.92 for ratios of 0.1 and above. Similarly, DBI maintained an accuracy of 0.92 for ratios of 0.4 and above. Nonetheless, its accuracy of 0.85 at a ratio of 0.1 is much lower than that of the BRI and BRIX variants.

**Table 5:** Results of the proposed methods (DBI, BRI & BRIX) on the USPS dataset with different reduction ratios.

Ratio	Training Speedup			Testing Speedup			Accuracy			Number of SVs			Jaccard similarity		
Whole dataset	1.00			1.00			0.922			790			1.00		
	DBI	BRI	BRIX	DBI	BRI	BRIX	DBI	BRI	BRIX	DBI	BRI	BRIX	DBI	BRI	BRIX
0.01	88.81	90.70	98.19	6.24	8.81	9.44	0.488	0.305	0.786	82	58	52	0.08	0.07	0.02
0.02	66.88	64.54	72.90	4.37	5.06	6.77	0.548	0.701	0.896	139	113	80	0.12	0.09	0.04
0.03	58.51	62.66	63.79	3.92	4.22	5.85	0.564	0.802	0.909	178	161	102	0.15	0.10	0.04
0.04	54.48	50.28	62.17	3.29	3.41	5.78	0.622	0.871	0.915	217	199	109	0.18	0.12	0.05
0.05	39.06	45.14	51.77	2.52	3.20	5.12	0.684	0.891	0.917	254	227	123	0.20	0.12	0.05
0.1	24.22	27.71	33.35	1.70	2.38	4.84	0.850	0.917	0.920	405	306	142	0.25	0.15	0.07
0.2	13.03	14.96	18.85	1.79	2.38	4.38	0.911	0.921	0.921	412	309	159	0.29	0.19	0.09
0.3	9.08	9.15	12.87	1.76	2.19	4.15	0.917	0.921	0.921	415	326	179	0.33	0.23	0.12
0.4	6.73	6.57	8.20	1.85	2.16	3.20	0.919	0.921	0.921	421	345	225	0.34	0.26	0.17
0.5	4.99	5.14	6.57	1.80	2.17	3.00	0.920	0.922	0.921	418	362	248	0.35	0.29	0.20
0.6	4.21	4.38	5.05	1.81	2.09	2.77	0.920	0.922	0.922	406	374	272	0.36	0.32	0.23
0.7	3.44	3.31	3.75	1.83	1.90	2.39	0.921	0.922	0.921	403	388	307	0.38	0.35	0.27
0.8	2.72	3.01	2.84	1.78	1.87	2.09	0.921	0.922	0.922	407	397	345	0.39	0.37	0.32
0.9	2.34	2.52	2.37	1.74	1.78	1.97	0.922	0.922	0.922	411	405	383	0.41	0.40	0.38

Each row compares performance metrics of the proposed methods for a different value (0.01 to 0.05 and 0.1 to 0.9) of the reduction ratio parameter *r* (the user-defined desired proportion of the training set to retain). The first row represents the metrics measured after training on the whole dataset.

**Fig 12:**
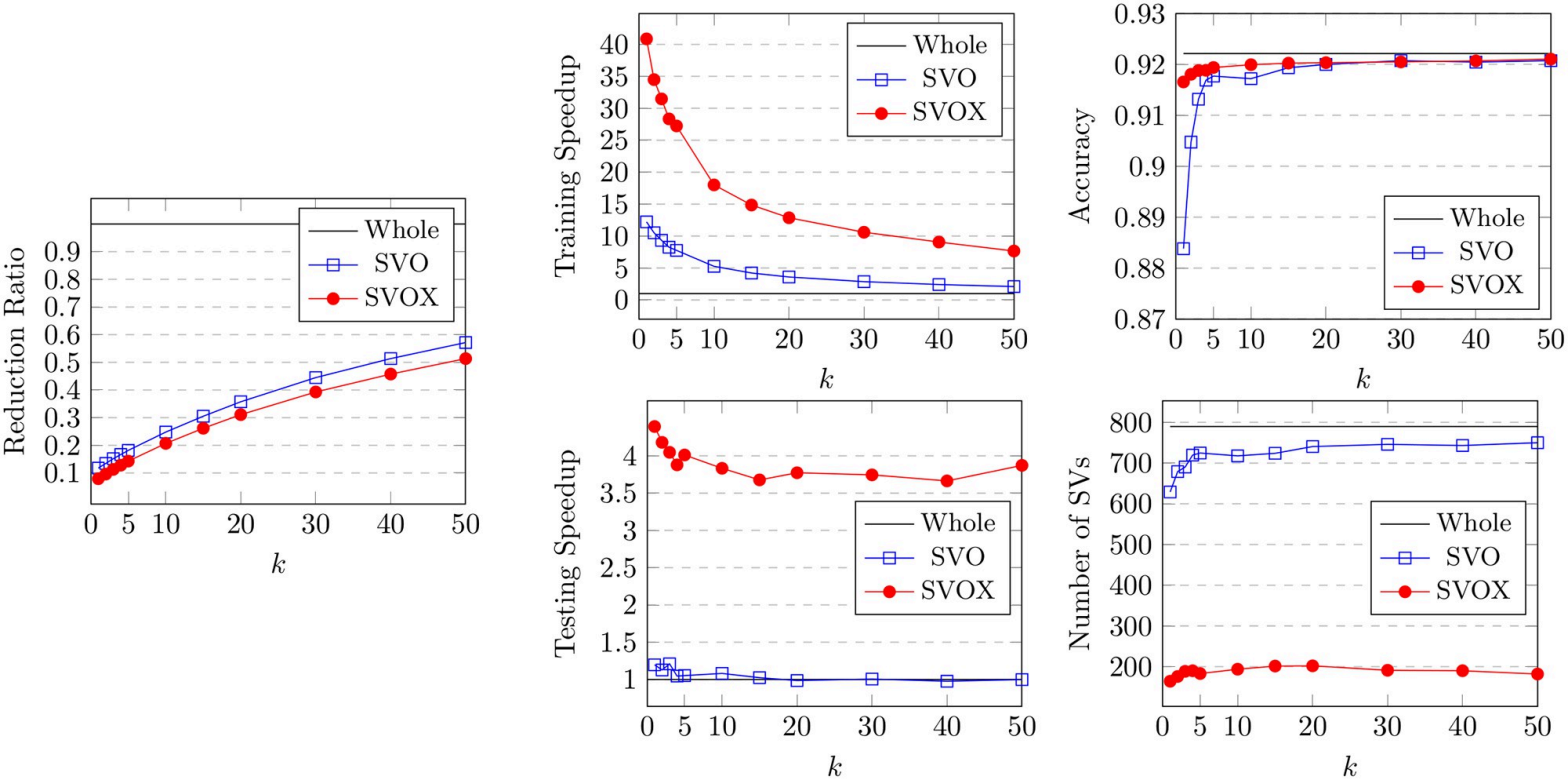
Results for the proposed SVO and SVOX methods on the USPS dataset with different values of *k*.

To evaluate the effectiveness of the SVO and SVOX proposed variants for high-dimensional datasets, we compared their performance for the USPS dataset. The results are presented in Fig 12 and Table 6.

**Table 6:** Results of the proposed SVO & SVOX methods on the USPS dataset with different values of *k*.

*k*	Reduced (ratio)		Training Speedup		Testing Speedup		Accuracy		Number of SVs		Jaccard similarity	
Whole dataset	1.00		1.00		1.00		0.922		790		1.00	
	SVO	SVOX	SVO	SVOX	SVO	SVOX	SVO	SVOX	SVO	SVOX	SVO	SVOX
1	0.12	0.08	12.22	40.87	1.20	4.39	0.884	0.917	629	164	0.39	0.11
2	0.13	0.10	10.51	34.48	1.13	4.18	0.905	0.918	679	176	0.40	0.12
3	0.15	0.11	9.33	31.47	1.21	4.05	0.913	0.919	690	189	0.40	0.13
4	0.17	0.13	8.25	28.34	1.05	3.88	0.917	0.919	720	190	0.41	0.13
5	0.18	0.14	7.76	27.24	1.05	4.01	0.918	0.919	725	183	0.41	0.13
10	0.25	0.21	5.26	18.01	1.08	3.83	0.917	0.920	718	194	0.46	0.14
15	0.31	0.26	4.22	14.86	1.02	3.68	0.919	0.920	724	202	0.49	0.15
20	0.36	0.31	3.59	12.86	0.99	3.77	0.920	0.920	741	202	0.50	0.15
30	0.44	0.39	2.87	10.60	1.01	3.75	0.921	0.920	746	191	0.53	0.15
40	0.51	0.46	2.41	9.06	0.98	3.66	0.920	0.921	743	190	0.57	0.16
50	0.57	0.51	2.10	7.66	1.00	3.87	0.921	0.921	750	182	0.58	0.15

Each row compares performance metrics of the methods for a different value (1 to 50) of the parameter *k* of the SVO & SVOX methods. The first row represents the metrics measured after training on the whole dataset. “Reduced (ratio)” is the ratio of the size of the resulting reduced dataset to the size of the whole dataset.

Both variants were tested against values for the *k* parameter, specifically 1, 2, 3, 4, 5, 10, 20, 30, 40, and 50. For SVOX, *N* and *L* were fixed at 15 and 0.5 respectively. It is to be noted that *k* includes the support vector itself, so the actual neighbor count to be included is *k* − 1. This means that for SVO, when *k* = 1, the reduced dataset will include only the support vectors identified by the SVC in 2 dimensions. For SVOX, *k* − 1 will include at most all the support vectors, since those with impure neighborhoods are excluded. It is then possible to evaluate the effect of the two strategies of adding neighbors of support vectors to the reduced dataset by comparison. SVOX shows a higher training time speedup of 40.8 at *k* = 1, compared to 12.2 for SVO. The speedup declines increasingly slower for larger *k* for both variants. SVOX also shows a higher accuracy at lower *k* values than SVO, as it starts at a value of 0.917 at *k* = 1 compared to 0.88 for SVO and a baseline of 0.922. This signifies the effect of SVOX’s strategy of pure-neighbor selection on enhancing the model’s accuracy. Both variants maintain a relatively stable testing speedup as *k* increases. Nevertheless, SVOX shows more than 3 times the speedup of SVO for both testing and training. The number of identified support vectors when using the reduced dataset obtained by SVOX is also significantly lower than that of SVO. SVOX contributed to identifying only 164 support vectors at *k* = 1 compared to 629 for SVO and a baseline of 790. The reduced dataset ratio is slightly lower for SVOX but increases almost linearly as *k* increases. It starts at 0.08 for SVOX at *k* = 1, while SVO shows a value of 0.12. These observations suggest that SVOX can identify a better subset of informative data points at very low reduction ratios.

Additionally, the proposed methods were compared experimentally to three existing methods from the literature: CBCH, BPLSH, and Shell Extraction (SE). The results are presented in Table 7. The parameters considered for the compared existing methods are as follows: CBCH with *k* ∈ {50, 100, …, 700}, BPLSH with *M* ∈ {10, 20, 30} and *L* ∈ {10, 30, 50, 70, 90, 110}, and SE with preservation ratios *r* ∈ {0.1, 0.2, …, 0.9}. Notably, due to technical limitations related to the memory requirements of constructing the convex hulls in dimensions higher than 8, the CBCH method was applied to the USPS dataset using the 2-dimensional UMAP embeddings, for instance selection, before training the SVM classifier trained on the corresponding selected subset in the 64-dimensional embeddings of the dataset, similar to the technique used for our proposed methods. For this comparison, the record selected for each method is the one with the minimum reduced dataset ratio and an accuracy of not less than 0.02 from the baseline classifier. In other words, the records are not necessarily the ones with the highest attainable accuracy for each method but rather the ones with maximum data reduction while maintaining acceptable accuracy.

**Table 7:** Results of the proposed methods compared to other methods from the literature on the USPS dataset.

Method		Reduced (ratio)	Accuracy	Training Speedup	Testing Speedup	Reduced SVs (ratio)
**Whole dataset**		1.00	0.922123	1.00	1.00	1.00
**CBCH (*k* = 400) [15]**		0.63182005	**0.922073**	1.621575	1.025486	0.961014
**BPLSH (*M* = 30;*L* = 20) [16]**		0.36004663	0.921923	3.254135	1.128958	0.857655
**Shell Extraction (SE) [14]**		0.4	0.918934	3.209440	1.118775	0.871665
**Proposed Methods**	**DBI (*r* = 0.2)**	0.2	0.911061	13.034276	1.785425	0.522033
	**BRI (*r* = 0.1)**	0.1	0.917389	27.714068	2.379853	0.387920
	**BRIX (*r* = 0.04)**	**0.04**	0.914898	**62.172803**	**5.783704**	**0.138205**
	**SVO (*k* = 3)**	0.15165272	0.913154	9.330182	1.209709	0.874003
	**SVOX (*k* = 1)**	0.07924839	0.916542	40.865746	4.393510	0.208468

The first row represents the metrics measured after training on the whole dataset. “Reduced (ratio)” is the ratio of the size of the resulting reduced dataset to the size of the whole dataset. “Reduced SVs (ratio)” is the ratio of the number of SVs of the reduced dataset to that of the whole dataset.

The results show that the proposed methods generally surpass the existing methods in terms of data reduction and speedup. Specifically, the BRIX method stands out as the most efficient, reducing the dataset to a mere 4% of its original size and achieving a training speedup of over 62 times and a testing speedup of 5.78. CBCH reduces the dataset to 63.18% of its original size, achieving almost the same accuracy as the baseline (0.922). BPLSH reduces the dataset to 36%, achieving slightly lower accuracy but faster training and testing processes. SE reduces the dataset to 40%, achieving slightly lower accuracy but faster training and testing processes. The proposed methods tend to retain a lower SV ratio compared to the existing methods. Specifically, the BRIX and SVOX methods preserve only 14% and 21% of the original SV count, respectively. In contrast to the proposed methods, the existing methods tend to retain a higher SV ratio. Specifically, CBCH, BPLSH, and SE preserve 96%, 86%, and 87% of the original SV count, respectively. In terms of accuracy, the proposed methods demonstrate comparable performance, with minor decreases from the baseline despite significant data reduction. This suggests that all methods effectively retain the essential information in the data for SVM training.

**Fig 13:**
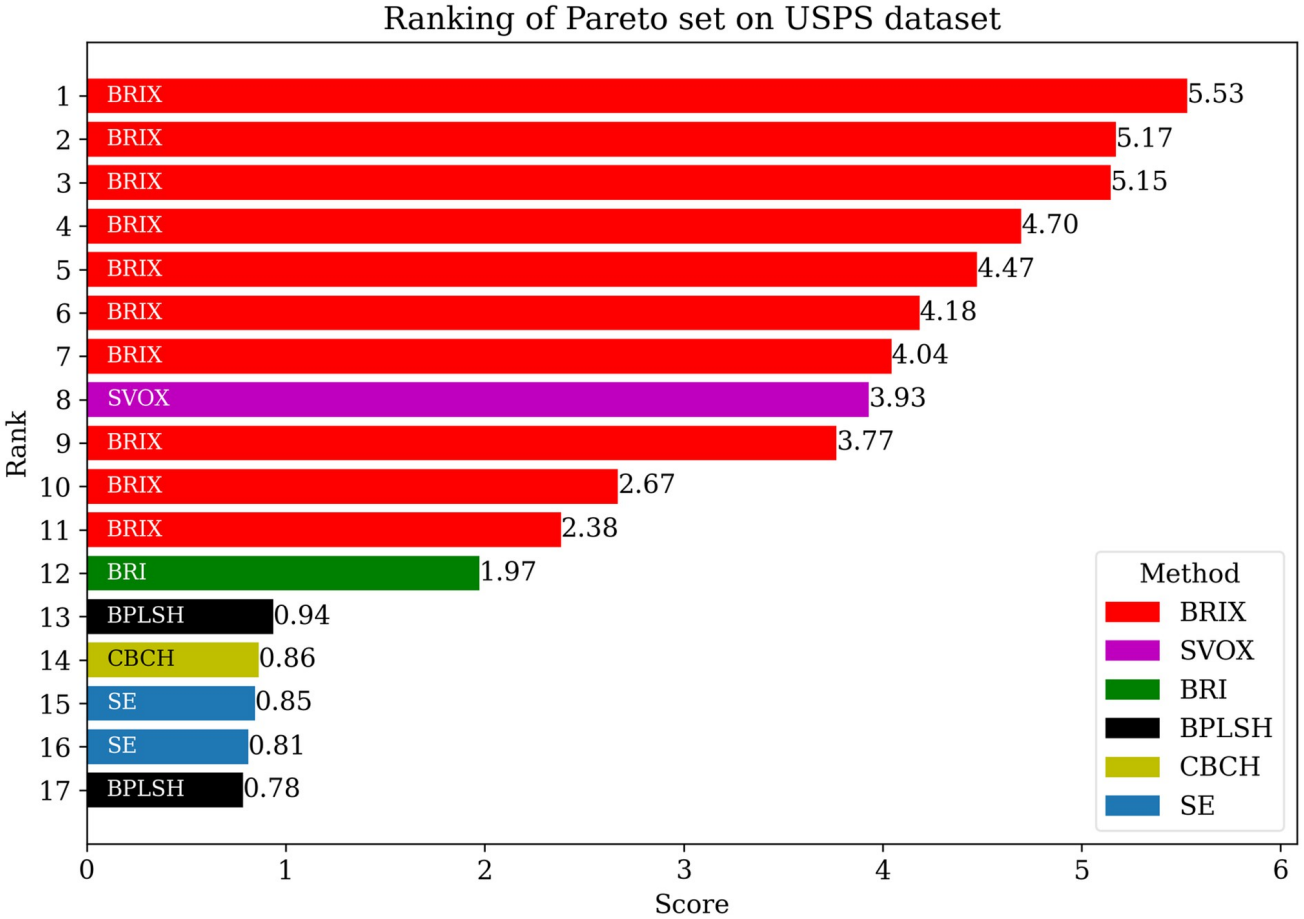
Ranking of Pareto set methods on the USPS dataset based on closeness to the optimal point. The optimal point is the zero point, representing the ideal of minimizing all the optimized metrics. The score is calculated as the reciprocal of the Euclidean distance from the optimal point.

The Pareto set was additionally identified for the proposed and compared methods for the USPS dataset. The list of Pareto set elements and their ranking are presented in Table 8 and Fig 13, respectively. The Pareto set is composed of 17 elements selected from 106 candidate solutions. BRIX dominates the Pareto set with 10 out of 17 elements, including the top 7 ranks. This indicates that BRIX is the most effective method in terms of the trade-off between accuracy, training time, and testing time.

**Table 8:** Pareto set of different methods on the USPS dataset.

Method	Reduced (ratio)	Accuracy	Training Speedup	Testing Speedup	Reduced SVs (ratio)	Rank
BRIX (*r* = 0.02)	0.02	0.896	72.90	6.77	0.10	1
BRIX (*r* = 0.04)	0.04	0.915	62.17	5.78	0.14	2
BRIX (*r* = 0.03)	0.03	0.909	63.79	5.85	0.13	3
BRIX (*r* = 0.05)	0.05	0.917	51.77	5.12	0.16	4
BRIX (*r* = 0.1)	0.10	0.920	33.35	4.84	0.18	5
BRIX (*r* = 0.01)	0.01	0.786	98.19	9.44	0.07	6
BRIX (*r* = 0.2)	0.20	0.921	18.85	4.38	0.20	7
SVOX (*k* = 2)	0.10	0.918	34.48	4.18	0.22	8
BRIX (*r* = 0.3)	0.30	0.921	12.87	4.15	0.23	9
BRIX (*r* = 0.5)	0.50	0.921	6.57	3.00	0.31	10
BRIX (*r* = 0.6)	0.60	0.922	5.05	2.77	0.34	11
BRI (*r* = 0.5)	0.50	0.922	5.14	2.17	0.46	12
BPLSH (*M* = 50;*L* = 20)	0.63	0.922	1.64	1.14	0.92	13
CBCH (*k* = 700)	0.67	0.922	1.54	1.05	0.96	14
SE (*r* = 0.7)	0.70	0.922	1.46	1.04	0.97	15
SE (*r* = 0.8)	0.80	0.922	1.30	1.04	0.98	16
BPLSH (*M* = 90;*L* = 30)	0.83	0.922	1.20	1.04	0.96	17

Methods in the Pareto set are ordered by their rank according to closeness to the optimal solution. “Reduced (ratio)” is the ratio of the size of the resulting reduced dataset to the size of the whole dataset. “Reduced SVs (ratio)” is the ratio of the number of SVs of the reduced dataset to that of the whole dataset.

**Fig 14:**
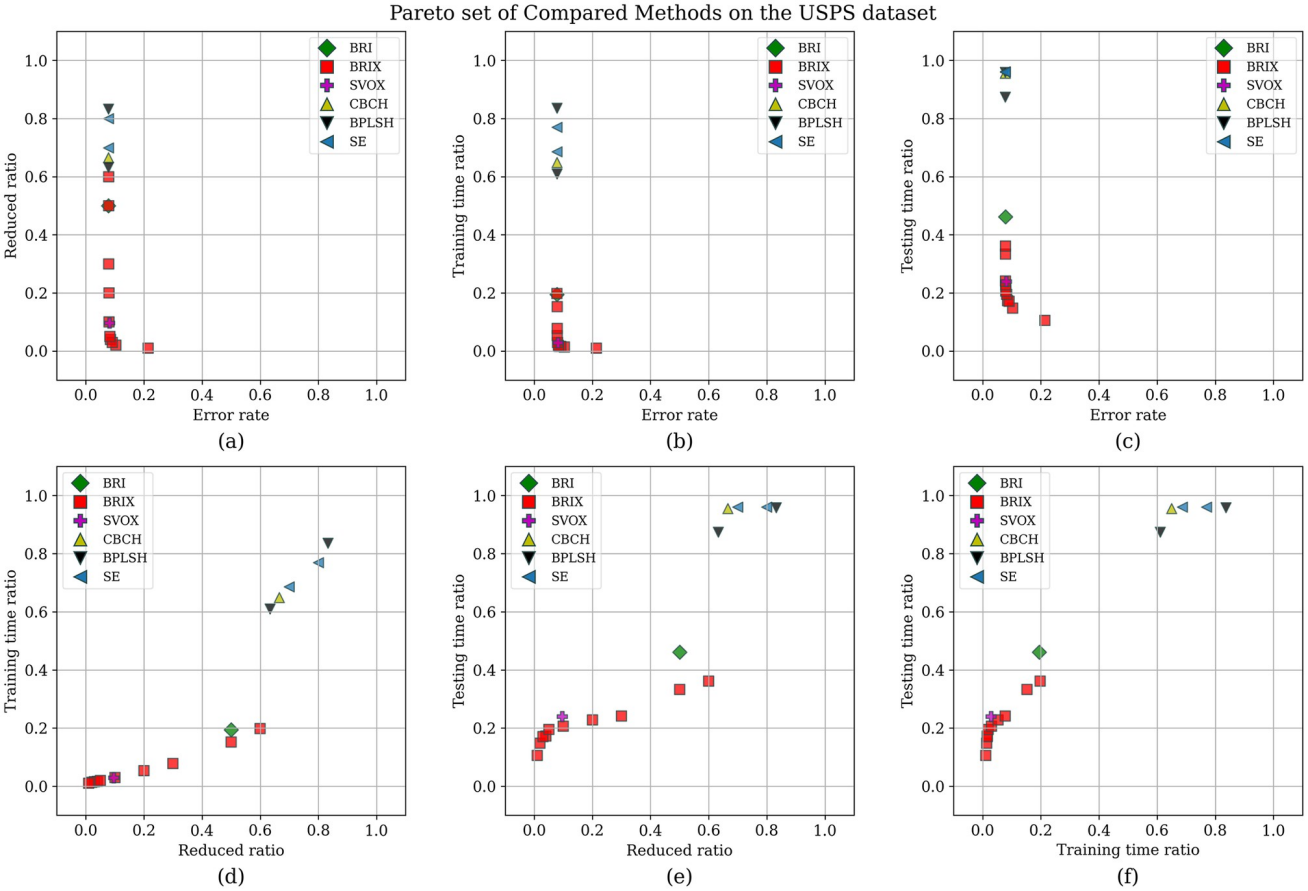
Pareto set for the USPS dataset. The Pareto set is composed of 17 elements selected from 106 candidate solutions.

**Fig 15:**
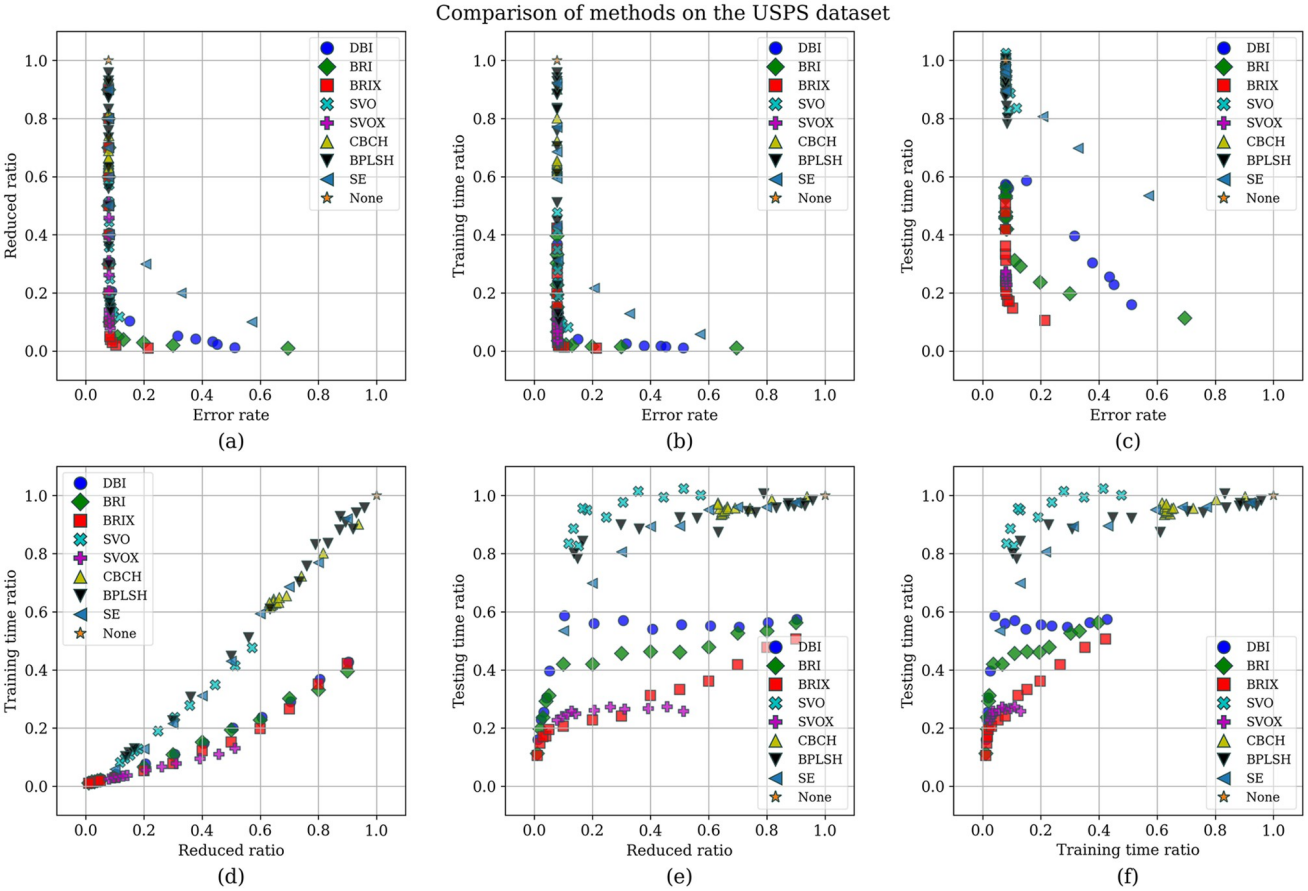
Comparison of methods on the USPS dataset. The distribution of the different methods in the solution space of the three optimization objectives, in addition to the ratio of the reduced dataset, is shown for each pair of objectives. The proposed methods, except SVO, are predominantly closest to the optimal point.

The Pareto set for the USPS dataset is presented in Fig 14. It can be observed that BRIX predominated other Pareto set elements in the regions closest to the optimal point for all the objectives. Furthermore, a wide gap is observed between the proposed methods and the compared methods in terms of all the objectives, where the proposed methods are grouped closer to the optimal point. Comparisons of the different methods in the solution space of different trade-off objectives are presented in Fig 15. It is evident that all the proposed methods except SVO predominantly occupy the closest regions to the optimal point and are superior in terms of testing time ratios, as they exclusively occupy the testing time ratios below 0.5. It is interesting to note that SVO is the only proposed method that has a similar distribution to the compared methods in the solution space of the three optimization objectives. Another interesting observation is that although BRIX, SVOX, BRI, and DBI show an almost similar linear correlation between the reduced dataset ratio and the training time ratio, BRIX and SVOX show a much lower testing time ratio for the same reduced dataset ratio. This is attributed to the significantly lower number of support vectors identified by BRIX and SVOX compared to BRI and DBI.

#### Adult9a

**Fig 16:**
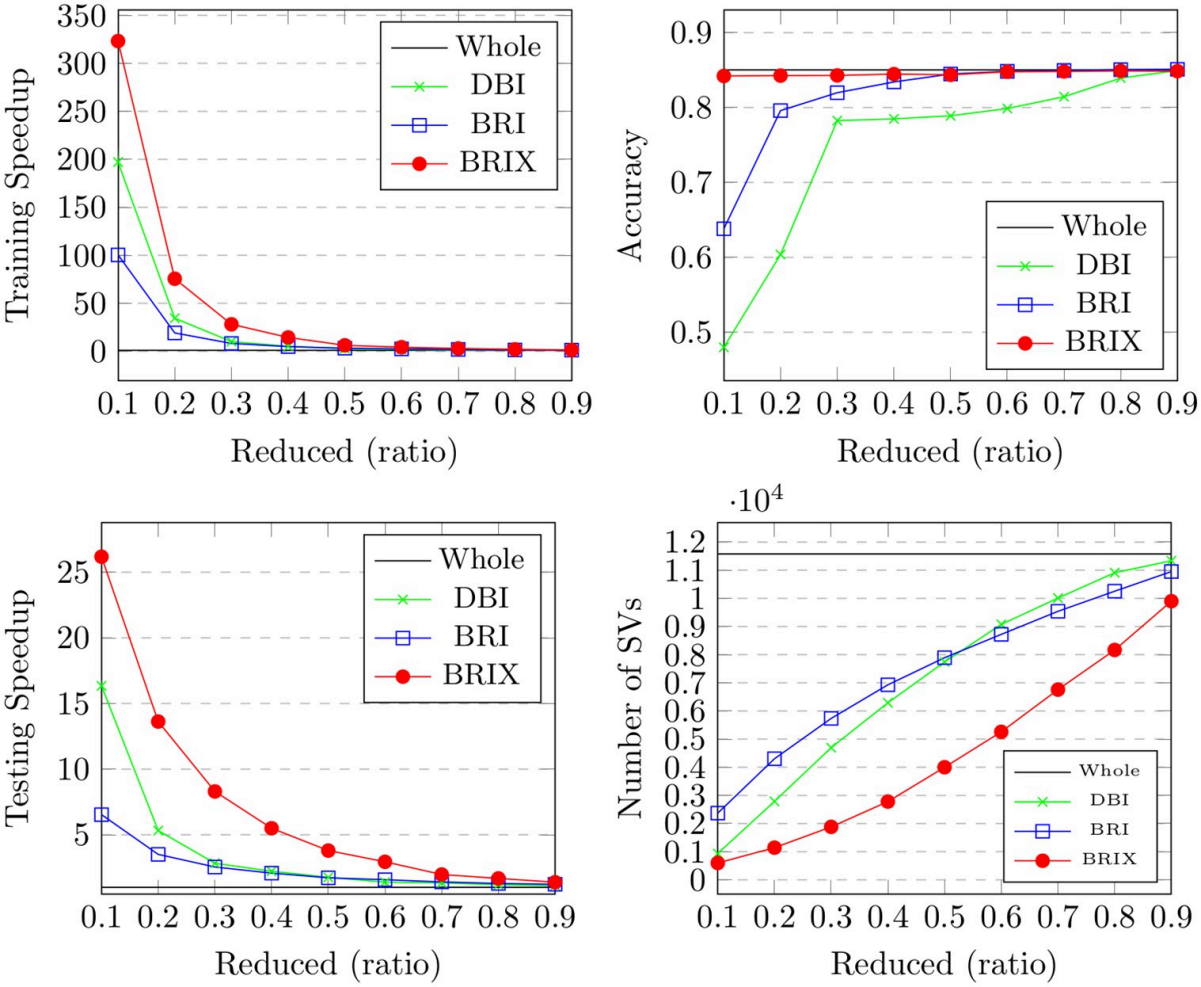
Results for our proposed methods (DBI, BRI, and BRIX) on the Adult9a dataset with different reduction ratios.

To assess our methods on imbalanced datasets, we employed our experiments on the Adult9a dataset. The outcomes are shown in Fig 16 and Table 9.

**Table 9:** Results of the proposed methods (DBI, BRI & BRIX) on the Adult9a dataset with different reduction ratios.

Ratio	Training Speedup			Testing Speedup			Accuracy			Number of SVs			Jaccard similarity		
Whole dataset	1.0			1.0			0.850			11580			1.00		
	DBI	BRI	BRIX	DBI	BRI	BRIX	DBI	BRI	BRIX	DBI	BRI	BRIX	DBI	BRI	BRIX
0.01	4568.5	9714.9	5732.3	173.1	251.1	148.5	0.437	0.182	0.805	78	52	89	0.00	0.00	0.01
0.02	2289.9	2251.4	2599.1	87.5	88.0	85.9	0.472	0.201	0.834	167	170	167	0.01	0.01	0.01
0.03	1865.6	711.8	1324.2	59.4	36.0	66.4	0.442	0.277	0.833	242	427	228	0.01	0.03	0.02
0.04	1265.2	347.5	1248.6	51.9	22.5	53.3	0.435	0.348	0.838	291	704	277	0.02	0.05	0.02
0.05	933.1	213.7	790.5	41.5	16.1	47.7	0.436	0.412	0.840	360	970	316	0.02	0.07	0.03
0.1	197.1	100.4	323.2	16.3	6.5	26.2	0.480	0.638	0.842	935	2371	599	0.06	0.15	0.05
0.2	34.4	19.1	75.6	5.3	3.5	13.6	0.604	0.796	0.843	2788	4302	1143	0.18	0.28	0.09
0.3	10.1	8.2	28.1	2.8	2.5	8.3	0.782	0.820	0.843	4695	5735	1882	0.30	0.36	0.15
0.4	5.1	4.9	14.4	2.2	2.1	5.5	0.785	0.834	0.845	6290	6929	2780	0.41	0.48	0.23
0.5	2.8	3.2	6.2	1.8	1.7	3.8	0.789	0.845	0.844	7742	7892	4000	0.52	0.56	0.32
0.6	1.9	2.5	4.3	1.4	1.6	2.9	0.799	0.848	0.848	9076	8721	5258	0.63	0.64	0.44
0.7	1.5	2.0	2.9	1.3	1.4	2.0	0.814	0.849	0.848	10010	9541	6758	0.67	0.73	0.56
0.8	1.6	1.4	2.0	1.2	1.3	1.7	0.839	0.851	0.849	10911	10254	8166	0.82	0.80	0.69
0.9	1.2	1.2	1.4	1.1	1.2	1.4	0.849	0.851	0.849	11336	10953	9900	0.89	0.90	0.82

Each row compares performance metrics of the proposed methods for a different value (0.01 to 0.05 and 0.1 to 0.9) of the reduction ratio parameter *r* (the user-defined desired proportion of the training set to retain). The first row represents the metrics measured after training on the whole dataset.

**Fig 17:**
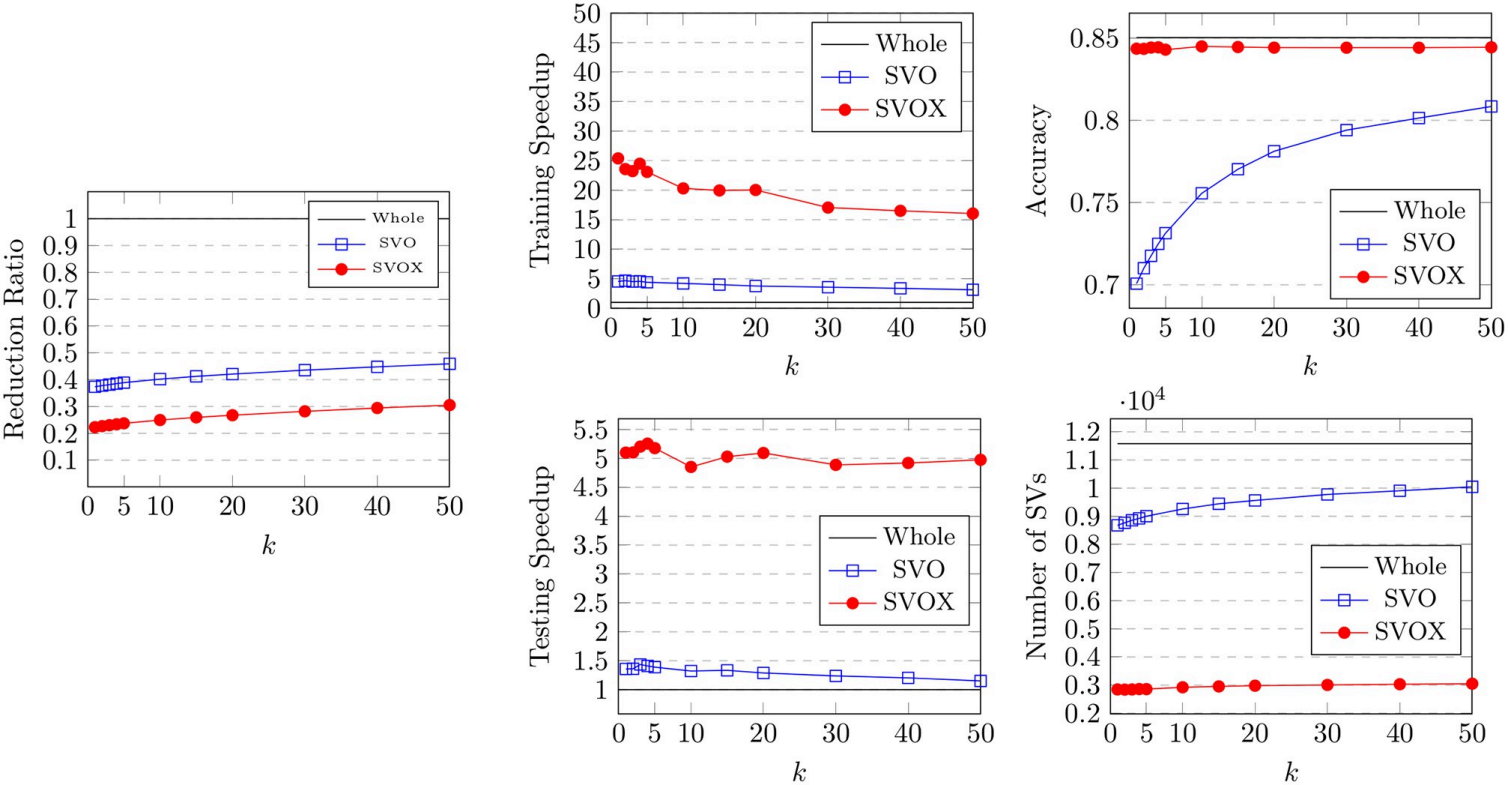
Results for the proposed SVO and SVOX methods on the Adult9a dataset with different values of *k*.

Consistent with findings from the other two datasets, it is observed that BRIX achieves the highest scores on all metrics, with a reduced ratio as low as 0.05, while achieving an acceptable accuracy of 0.840, less than 1% below the baseline (0.850), a training speedup of 790, a testing speedup of 47, and 0.03 of the baseline SV count. BRI ranks second, but only for ratios of 0.5 and above, where its accuracy is at or above 0.845. Similar to the results for Banana, the increase in SV counts is nearly linear with growing ratios. In contrast to other datasets, DBI’s precision is significantly diminished, which may indicate that its selection strategy is less suited for imbalanced datasets. In contrast, the BRIX strategy is more resistant to imbalanced datasets. SVO and SVOX were also evaluated against the Adult9a dataset. The findings are presented in Fig 17 and Table 10. Similar to the previous datasets, SVOX shows a higher training speedup of about 5 times that of SVO, starting at 25 at *k* = 1 and slowly declining to reach 16 at *k* = 50. SVOX also achieves a testing speedup of about 5 times that of SVO, starting at 5.1 at *k* = 1 and declining to 4.9 at *k* = 50. The most prominent observation is that SVOX maintains an almost constant accuracy of around 0.844 for all tested values of *k*, while, similar to DBI, SVO shows a significantly lower accuracy of 0.7 at *k* = 1, which rises with an increasingly slower rate to reach 0.8 at *k* = 50. The number of identified support vectors is also significantly lower for SVOX, accounting for 0.24 of the baseline at *k* = 1. Both variants show an extremely slow increase in the number of SVs and the ratio of the reduced dataset as *k* increases. This may be due to the increasing probability of including shared neighbors and other support vectors with increasing values of *k*. These findings may suggest that SVOX is more effective in identifying a better subset of informative data points at very low reduction ratios for imbalanced datasets.

**Table 10:** Results of the proposed SVO & SVOX methods on the Adult9a dataset with different values of *k*.

*k*	Reduced (ratio)		Training Speedup		Testing Speedup		Accuracy		Number of SVs		Jaccard similarity	
Whole dataset	1.00		1.00		1.00		0.850		11580		1.00	
	SVO	SVOX	SVO	SVOX	SVO	SVOX	SVO	SVOX	SVO	SVOX	SVO	SVOX
1	0.37	0.22	4.54	25.38	1.36	5.10	0.701	0.844	8678	2845	0.58	0.24
2	0.38	0.23	4.67	23.57	1.36	5.10	0.710	0.843	8768	2839	0.59	0.24
3	0.38	0.23	4.52	23.23	1.44	5.20	0.718	0.844	8858	2845	0.60	0.24
4	0.39	0.23	4.55	24.47	1.41	5.25	0.725	0.844	8925	2859	0.60	0.24
5	0.39	0.24	4.40	23.09	1.39	5.18	0.731	0.843	9003	2857	0.61	0.24
10	0.40	0.25	4.20	20.30	1.32	4.85	0.756	0.845	9259	2923	0.63	0.25
15	0.41	0.26	4.00	19.95	1.33	5.03	0.770	0.845	9445	2953	0.65	0.25
20	0.42	0.27	3.76	20.02	1.29	5.09	0.781	0.844	9562	2979	0.66	0.25
30	0.44	0.28	3.56	17.05	1.24	4.89	0.794	0.844	9775	3006	0.68	0.25
40	0.45	0.29	3.37	16.50	1.20	4.92	0.801	0.844	9907	3031	0.69	0.26
50	0.46	0.31	3.13	16.04	1.15	4.97	0.808	0.844	10047	3049	0.71	0.26

Each row compares performance metrics of the methods for a different value (1 to 50) of the parameter *k* of the SVO & SVOX methods. The first row represents the metrics measured after training on the whole dataset. “Reduced (ratio)” is the ratio of the size of the resulting reduced dataset to the size of the whole dataset.

Our findings were compared to those of FIFDR [13], Gaffari’s method [17] and Shell Extraction (SE) [14] according to the literature [17]. Table 11 summarizes the comparison. For this comparison, the records for our proposed methods were selected such that they have the minimum reduced dataset ratio while attaining an accuracy of not less than 0.01 from the baseline classifier (0.850). BRIX outperforms the other methods in terms of reduced dataset ratio (0.05), training speedup (790), testing speedup (47) and SV count (0.03 of baseline), while Gaffari’s method ranked second with reported values of 0.15, 61.9, 21, and 0.08, respectively. SVOX ranks third with respect to the same metrics, with values of 0.22, 25, 5, and 0.25, respectively. FIFDR and DBI rank first with an accuracy of (0.849), followed by BRI and Gaffari’s method at (0.845) and SVOX at (0.844). DBI ranks last for SV count (0.98 of baseline) and reduced dataset ratio (0.9), preceded by FIFDR with a ratio of 0.52 and an SV count of 0.97. The results of Shell Extraction and SVO had accuracy values of 0.7 and 0.8, respectively, which may not be suitable for this dataset based on the specified accuracy threshold.

**Table 11:** Results of the proposed methods compared to other methods from the literature on the Adult9a dataset.

Method		Reduced (ratio)	Accuracy	Training Speedup	Testing Speedup	Reduced SVs (ratio)
**Whole dataset**		1.00	0.850	1.00	1.00	1.00
**FIFDR [13]**		0.523	**0.849**	2.1	1.023	0.974
**Gaffari [17]**		0.147	0.845	61.89	21.73	0.08
**Shell Extraction (SE) [14]**		0.374	0.703	7.5	3.43	0.361
**Proposed Methods**	**DBI (*r* = 0.9)**	0.9	**0.849477**	1.174875	1.137709	0.978957973
	**BRI (*r* = 0.4)**	0.4	0.844747	3.222566	1.724006	0.681548647
	**BRIX (*r* = 0.05)**	**0.05**	0.840448	**790.536832**	**47.744518**	**0.027288428**
	**SVO (*k* = 50)**	0.45924572	0.808396	3.131336	1.150444	0.867573402
	**SVOX (*k* = 1)**	0.22302755	0.843529	25.380079	5.098934	0.245682211

The first row represents the metrics measured after training on the whole dataset. “Reduced (ratio)” is the ratio of the size of the resulting reduced dataset to the size of the whole dataset. “Reduced SVs (ratio)” is the ratio of the number of SVs of the reduced dataset to that of the whole dataset.

**Fig 18:**
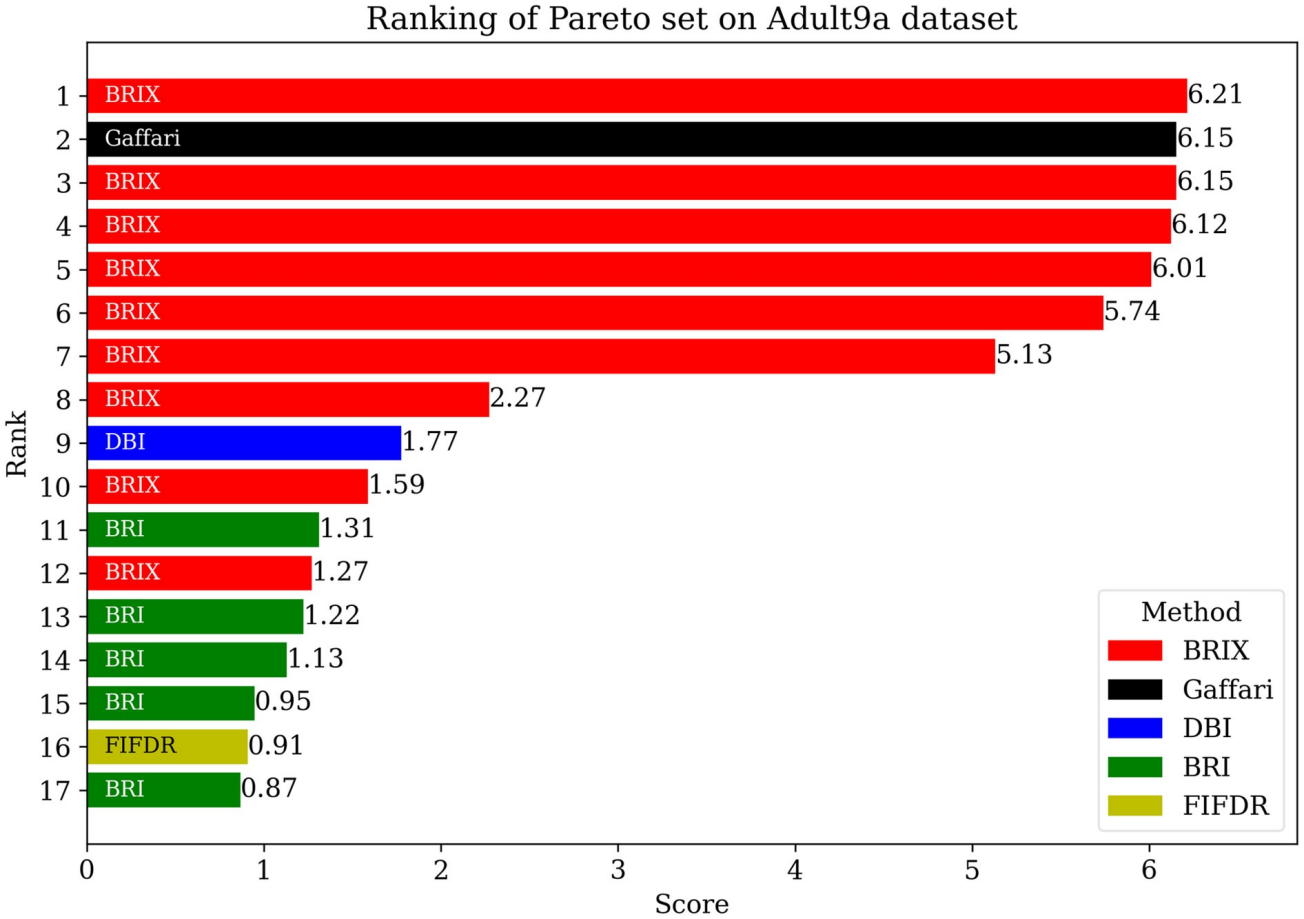
Ranking of Pareto set methods on the Adult9a dataset based on closeness to the optimal point.

Table 12 lists the Pareto set elements for the proposed and compared methods for the Adult9a dataset, and their ranking is presented in Fig 18. The Pareto set is composed of 17 elements selected out of 68 possible candidate solutions. BRIX ranks first, followed very closely by Gaffari’s method. BRI constitutes five elements of the set, however, with relatively lower scores than BRIX. SVO and SVOX were not included in the Pareto set.

**Table 12:** Pareto set of different methods on the Adult9a dataset.

Method	Reduced (ratio)	Accuracy	Training Speedup	Testing Speedup	Reduced SVs (ratio)	Rank
BRIX (*r* = 0.05)	0.05	0.840	790.54	47.74	0.03	1
Gaffari	0.15	0.845	61.89	21.73	0.08	2
BRIX (*r* = 0.1)	0.10	0.842	323.17	26.18	0.05	3
BRIX (*r* = 0.04)	0.04	0.838	1248.59	53.25	0.02	4
BRIX (*r* = 0.02)	0.02	0.834	2599.13	85.95	0.01	5
BRIX (*r* = 0.2)	0.20	0.843	75.57	13.62	0.10	6
BRIX (*r* = 0.01)	0.01	0.805	5732.26	148.46	0.01	7
BRIX (*r* = 0.6)	0.60	0.848	4.26	2.94	0.45	8
DBI (*r* = 0.01)	0.01	0.437	4568.53	173.11	0.01	9
BRIX (*r* = 0.7)	0.70	0.848	2.92	1.98	0.58	10
BRI (*r* = 0.6)	0.60	0.848	2.50	1.58	0.75	11
BRIX (*r* = 0.8)	0.80	0.849	2.03	1.68	0.71	12
BRI (*r* = 0.01)	0.01	0.182	9714.89	251.12	0.00	13
BRI (*r* = 0.7)	0.70	0.849	1.95	1.41	0.82	14
BRI (*r* = 0.8)	0.80	0.851	1.42	1.29	0.89	15
FIFDR	0.52	0.849	2.10	1.02	0.97	16
BRI (*r* = 0.9)	0.90	0.851	1.24	1.23	0.95	17

Methods in the Pareto set are ordered by their rank according to closeness to the optimal solution. “Reduced (ratio)” is the ratio of the size of the resulting reduced dataset to the size of the whole dataset. “Reduced SVs (ratio)” is the ratio of the number of SVs of the reduced dataset to that of the whole dataset.

**Fig 19:**
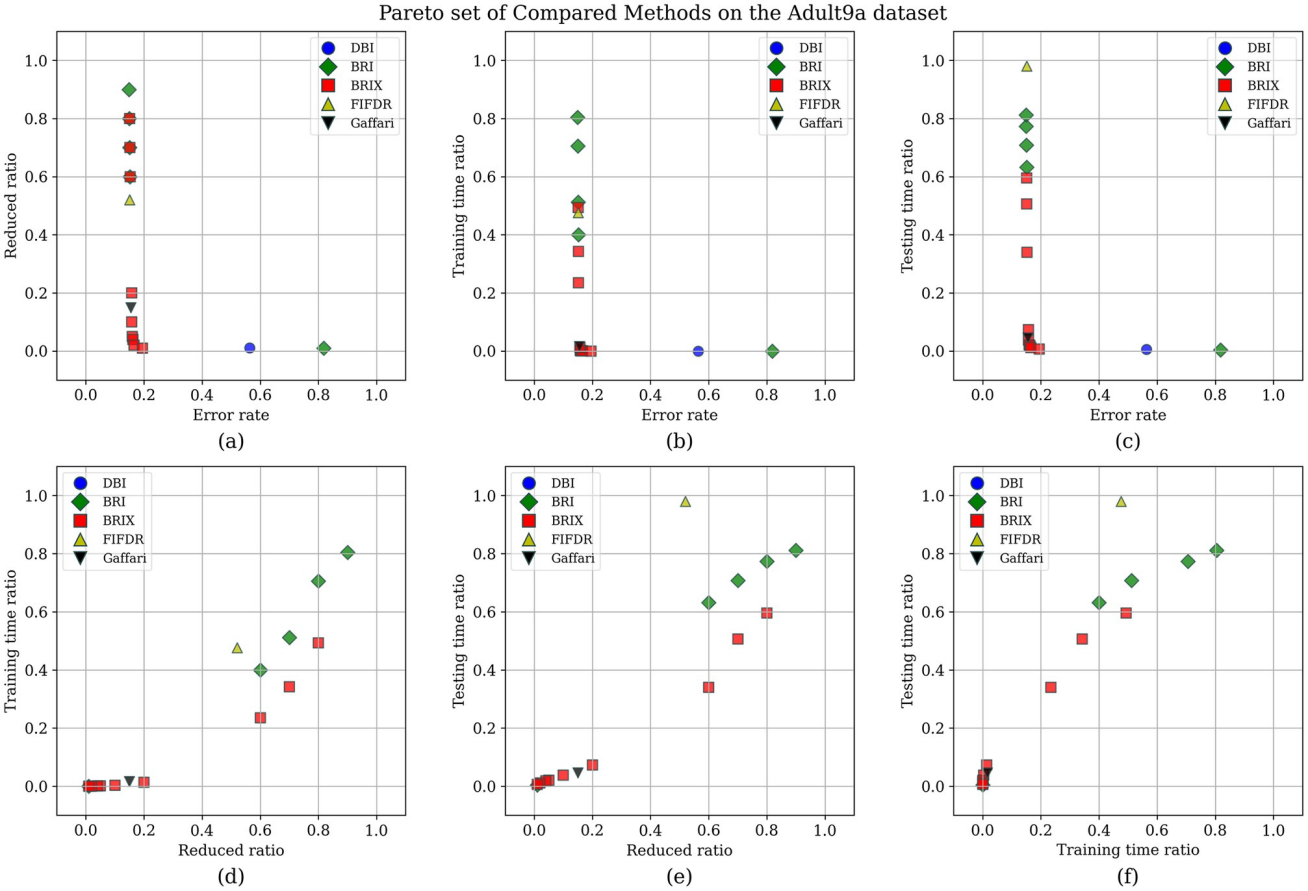
Pareto set for the Adult9a dataset. The Pareto set is composed of 17 elements selected out of 68 possible candidate solutions.

**Fig 20:**
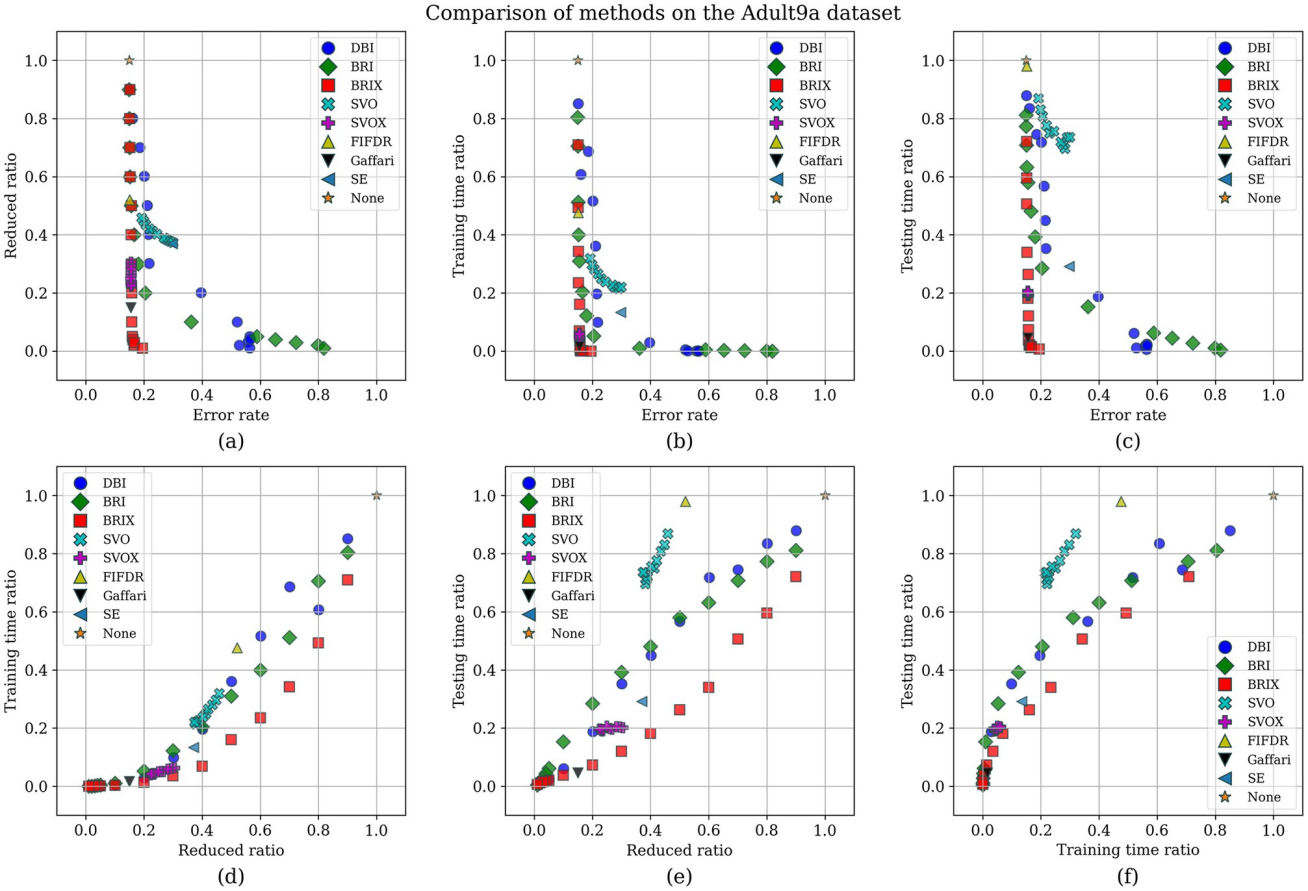
Comparison of methods on the Adult9a dataset. The distribution of the different methods in the solution space of the three optimization objectives, in addition to the ratio of the reduced dataset, is shown for each pair of objectives. BRIX and Gaffari’s method are the closest to the optimal point. SVO and SVOX are more clustered in the solution space than the other methods.

The Pareto set for the Adult9a dataset is presented in Fig 19. Consistent with the ranking of the Pareto set elements, BRIX and Gaffari’s method lie the closest to the optimal point for all the objectives. Furthermore, the top-ranking methods, namely BRIX and Gaffari’s method, are separated from the other methods with a wide gap in terms of all the objectives. This may indicate they have relatively similar efficiency on the Adult9a dataset. This may be attributed to the fact that Gaffari’s objective of reducing complexity at the decision boundary is, in a sense, similar to BRIX’s objective of selecting more points from non-overlapping regions. It is to be noted that all available points in the solution space were considered for inclusion in the Pareto set. This allowed the presence of points with extreme trade-offs, such as those for DBI and BRI, at a ratio of 0.01 where accuracy was sacrificed entirely in favor of extreme speedups, rendering them practically useless solutions that can be safely ignored. In a practical setting, a specific accuracy threshold may be specified so that points with accuracy below the threshold would not be considered for inclusion in the Pareto set. A comparison of the different methods in the solution space of different trade-off objectives is presented in Fig 20. It confirms the superiority of BRIX and Gaffari’s method over the other methods in terms of the optimization objectives. It is interesting to note that SVO and SVOX have a more clustered distribution in the solution space than the other methods. However, the cluster for SVOX is close to the optimal point, while that for SVO is relatively far from it. This may also indicate that SVOX is more effective in identifying a better subset of informative data points due to its similarity to BRIX in terms of the selection strategy.

For reference, recorded training and testing times for the proposed methods on USPS and Adult9a datasets can be found in S1 File.

## 5 Discussion

This study proposed and investigated the performance of five proposed instance selection methods, specifically three density-based methods (DBI, BRI, and BRIX) and two SVM-based methods (SVO and SVOX). The proposed methods were applied to three datasets: Banana, USPS, and Adult9a, to reduce dataset sizes before training SVM classifiers. The effectiveness of the proposed methods was assessed in terms of classification accuracy, training and testing speedups, reduced dataset ratio, and support vector count. Additionally, comparisons with other state-of-the-art methods were conducted.

The findings revealed that the proposed variants of the DBI method, especially BRIX and SVOX, effectively reduced the size of the training data and achieved significant training and prediction speedups while maintaining adequate classification accuracy compared to training on the original dataset. The proposed methods outperformed the related existing methods in terms of the extent of reduction, speedups, and support vector count. The proposed methods also demonstrated their usefulness on high-dimensional datasets by applying instance selection in lower-dimensional embeddings before mapping the reduced dataset back to the original higher-dimensionality for training.

Notably, the BRIX method stood out as the most efficient and effective method, achieving the highest scores on all metrics and dominating the Pareto sets for all datasets, indicating its superiority in balancing multiple objectives. It reduced the dataset to a very small fraction of its original size while conveniently approaching the accuracy levels of training the SVM on the full dataset. It also achieved significant speedups in both training and testing times and reduced the number of support vectors considerably.

The superiority of BRIX may be attributed to its selection strategy, which combines two different approaches to instance selection scoring. The first favors the selection of more points from boundary regions, which are more likely to contain candidate support vectors. The second discourages the selection of points from overlapping regions, which are less informative for decision boundary identification. This combination of strategies would result in the selection of a more informative subset of data points. It would also reduce the number of identified support vectors, resulting in a substantial speedup in both training and testing times. Furthermore, the use of weighted random sampling using the combined scoring scheme ensures that points from different regions of the dataset are also represented in the reduced dataset. This somewhat permissive selection strategy would help preserve the diversity of the dataset and improve the generalization of the trained model.

SVOX also showed promising results, which is possibly due to a similar selection strategy to BRIX, which selects support vectors and their neighbors only if their neighborhoods are dominated by the same class. Nevertheless, SVOX was outperformed by BRIX on all datasets, especially in terms of the number of identified support vectors and thus training and testing speedups. This may possibly be attributed to the inclusion of a specific number of neighbors for each support vector, which may include redundant points, especially in cases where many support vectors are close neighbors. This would increase the number of identified support vectors and thus reduce the speedups.

DBI and SVO were of modest performance and both struggled evidently on the Adult9a dataset, suggesting their selection strategies may be susceptible to class skew. DBI was the least effective, mainly due to its selection of points that are strictly at the borders of classes, which is not effective for datasets with significant overlap between classes, especially at lower ratios of the reduced dataset. SVO was also less effective than SVOX, which may be attributed to its selection of neighbors for support vectors regardless of their neighborhood properties. This may result in the selection of more redundant points in class-impure regions, which would increase the number of identified support vectors and further degrade the speedups. Moreover, the strict deterministic nature of the selection strategies of DBI, SVO, and SVOX may affect the generalization ability of the trained model by preventing the inclusion of any representative points from different regions of the dataset. BRI, on the other hand, shares a similarly permissive selection strategy with BRIX, which may explain its advantage over DBI and SVO.

These observations and their interpretations substantiate the effectiveness of prioritizing selection from non-overlapping regions in preserving class boundary information. Adopting this selection strategy aligns with Gaffari’s method [17], which aims at reducing the complexity of the decision boundary through the removal of harmful points (i.e., those with other classes dominating their neighborhoods). However, it contrasted with other methods such as CBCH [15] and BPLSH [16], where boundaries are identified based on impure-class neighborhoods. They also suggest that, for the proposed density-based methods, including additional points beyond the strict layer of border points was crucial for achieving acceptable accuracy levels, especially for smaller sizes of the reduced dataset. Furthermore, training times are influenced by the quality of the selected subset and its convenience to the SVM optimization algorithm, as evident from the different training speedups attained by different methods despite having similar reduced dataset ratios.

Another observation is that for all the proposed density-based methods, the number of identified support vectors would gradually increase as more points were retained until reaching the baseline count for the whole dataset. An unexpected exception to that was observed for the USPS dataset, where the support vector count did not exceed half of the baseline count for all reduced density-based variants. This suggests that support vector counts are guaranteed to drop by at least 50% for this dataset, regardless of the extent of reduction applied by the density-based selection algorithms. This observation warrants further investigation.

These findings demonstrate the feasibility of applying density-based techniques to significantly reduce training data sizes for SVMs to achieve substantial speed advantages. Moreover, the findings indicate that lower-dimensional embeddings produced by manifold-learning techniques can potentially help analyze the neighborhood properties of high-dimensional datasets and facilitate instance selection. The findings also emphasize the importance of carefully considering the trade-off between size reduction and accuracy to ensure optimal model performance for specific tasks and application contexts. The study also highlights the utility of controlling the reduced dataset ratio as a parameter to cover the trade-off space between speedup and accuracy. Furthermore, the evaluation methodology employed in this study, which considered multiple objectives simultaneously, helped reveal the diverse trade-offs among methods and provide a comprehensive comparison.

However, the study has some limitations, such as: the proposed methods were evaluated on a limited number of datasets; the proposed density-based methods assume that classes are densest at their centers, which may not hold for some datasets; and they are sensitive to the choice of the *ε* and *minPts* parameters, although this sensitivity is assumed to have been reduced by UMAP embeddings. Additionally, the performance of the proposed methods for high-dimensional datasets depends on the quality of the UMAP embeddings, which may vary for different datasets. Moreover, fixed parameters were used across classes for density-based methods, which may not be optimal for all classes.

Therefore, further research may include evaluating the proposed methods on a wider range of datasets, investigating the effect of the *ε* and *minPts* parameters on the performance of the proposed methods, and optimizing the parameters individually for each class. Additionally, the effectiveness of instance selection in lower-dimensional embeddings produced by other manifold-learning techniques may be investigated. Furthermore, the automatic selection of parameters for the proposed methods may be explored. Finally, the impact of data reduction on various types of SVM kernels may be analyzed.

## 6 Conclusion

In this research, we proposed a new density-based method for reducing SVM training data to speed up SVM training of large datasets in higher dimensions. Our approach uses UMAP to produce a lower-dimensional embedding of the data, where it extracts a subset in the form of a layer of border points based on scores calculated by analyzing their neighborhood properties. Subsets can be repeatedly mapped, using their indices, to the original dataset or any other higher-dimensional embedding for SVM training. We also introduced a modified k-fold cross-validation method that adapts to training on a subset of the dataset with a different distribution. Experimental findings on the selected datasets demonstrate that our approach variants BRIX and SVOX show the most promising results, achieving significant training and prediction speedups and considerable reductions in the size of the training set and the number of identified support vectors while maintaining almost similar accuracy levels compared to training the SVM on the full dataset. Comparisons to other methods in the literature support the effectiveness of our approach, particularly in terms of training and testing speedups and the number of identified support vectors. These findings suggest that our approaches for instance selection in lower-dimensional spaces, are relevant and thus beneficial for high-dimensional datasets.

## Supporting information

S1 File Tables for training and testing times of the proposed methods on USPS and Adult9a datasets. (PDF)

S2 File List of acronyms used in this paper. (PDF)
