# Supplementary material for: Data reduction for SVM training using density-based border identification
Source: PLoS One. 2024 Apr 3;19(4):e0300641. doi: 10.1371/journal.pone.0300641 (PMC10990207; doi:10.1371/journal.pone.0300641)
Supplement: S1 File — (PDF) [file pone.0300641.s001.pdf]

S1 File. Tables for training and testing times of the proposed methods on USPS and Adult9a datasets.

Table 1. Training and testing times of the proposed methods (DBI, BRI & BRIX) on the USPS dataset with different reduction ratios.

Ratio	Training time (s)			Testing time (s)		
Whole dataset	0.337			0.113		
	DBI	BRI	BRIX	DBI	BRI	BRIX
0.01	0.004	0.004	0.004	0.019	0.014	0.012
0.02	0.005	0.005	0.006	0.027	0.023	0.017
0.03	0.006	0.006	0.005	0.030	0.028	0.020
0.04	0.006	0.007	0.006	0.035	0.033	0.020
0.05	0.009	0.008	0.007	0.053	0.036	0.024
0.1	0.014	0.012	0.013	0.068	0.049	0.024
0.2	0.026	0.023	0.018	0.066	0.048	0.027
0.3	0.038	0.040	0.027	0.065	0.053	0.028
0.4	0.051	0.051	0.042	0.063	0.054	0.036
0.5	0.068	0.065	0.054	0.064	0.054	0.038
0.6	0.081	0.078	0.069	0.062	0.055	0.042
0.7	0.102	0.103	0.091	0.062	0.059	0.049
0.8	0.130	0.119	0.122	0.065	0.061	0.054
0.9	0.144	0.132	0.145	0.064	0.065	0.060

Table 2. Training and testing times of the proposed SVO & SVOX methods on the USPS dataset with different values of k.

k	Training time (s)		Testing time (s)	
Whole dataset	0.348		0.115	
	SVO	SVOX	SVO	SVOX
1	0.029	0.009	0.097	0.027
2	0.033	0.010	0.103	0.030
3	0.037	0.011	0.101	0.030
4	0.041	0.012	0.110	0.031
5	0.044	0.013	0.112	0.029
10	0.064	0.018	0.110	0.031
15	0.082	0.023	0.112	0.032
20	0.096	0.027	0.117	0.032
30	0.120	0.033	0.117	0.031
40	0.146	0.040	0.122	0.035
50	0.164	0.045	0.115	0.030

Table 3. Training and Testing times of the proposed methods (DBI, BRI & BRIX) on the Adult9a dataset with different reduction ratios.

Ratio	Training time (s)			Testing time (s)		
Whole dataset	86.861			25.665		
	DBI	BRI	BRIX	DBI	BRI	BRIX
0.01	0.03	0.01	0.02	0.30	0.21	0.31
0.02	0.02	0.06	0.02	0.47	0.46	0.47
0.03	0.05	0.09	0.05	0.67	0.86	0.58
0.04	0.08	0.15	0.08	0.86	1.24	0.74
0.05	0.11	0.24	0.10	0.99	1.73	0.91
0.1	0.38	0.70	0.28	2.06	4.33	1.37
0.2	1.82	2.78	1.06	4.83	8.23	2.70
0.3	5.74	6.31	2.66	8.62	11.87	3.95
0.4	11.42	12.26	5.75	12.49	14.76	5.72
0.5	18.25	19.32	10.26	15.69	15.67	8.35
0.6	26.87	27.24	16.00	19.06	17.77	10.47
0.7	35.56	35.69	25.49	20.59	20.72	14.39
0.8	51.58	48.34	40.39	24.20	20.66	17.31
0.9	67.50	66.32	60.72	25.54	24.04	22.21

Table 4. Training and testing times of the proposed SVO & SVOX methods on the Adult9a dataset with different values of k.

k	Training time (s)		Testing time (s)	
Whole dataset	83.503		26.129	
	SVO	SVOX	SVO	SVOX
1	13.809	2.302	15.954	5.294
2	14.635	2.514	16.456	5.425
3	14.865	2.486	17.058	5.396
4	14.875	2.464	16.444	5.249
5	14.958	2.626	16.438	5.363
10	16.387	2.980	17.695	5.439
15	17.083	3.133	18.318	5.609
20	17.844	3.176	17.657	5.720
30	19.280	3.725	18.744	5.785
40	20.286	3.848	19.488	5.869
50	21.197	3.989	19.194	5.984
