# Supplementary material for: Data reduction for SVM training using density-based border identification
Source: PLoS One. 2024 Apr 3;19(4):e0300641. doi: 10.1371/journal.pone.0300641 (PMC10990207; doi:10.1371/journal.pone.0300641)
Supplement: S2 File — (PDF) [file pone.0300641.s002.pdf]

S2 File. List of Acronyms used in this paper.

Notation	Description
BPLSH	border point extraction based on locality-sensitive hashing
BRI	Border-biased Random Instance selection
BRIX	Border-biased Random Instance selection with eXclusion
CBCH	clustering-based convex hull
DBI	Density-based Border Identification
DBSCAN	Density-Based Spatial Clustering of Applications with Noise
FDR	Fisher Discriminant Ratio
FIFDR	Fast Iteration of FDR
KM-SVM	K-means SVM
LSH	locality-sensitive hashing
PCA	Principal Components Analysis
RBF	Radial Basis Function
SE	Shell Extraction
SV	Support Vector
SVM	Support Vector Machine
SVO	Support Vector Oracle
SVOX	Support Vector Oracle with eXclusion
t-SNE	t-Distributed Stochastic Neighbor Embedding
UMAP	Uniform Manifold Approximation and Projection
